# Thriving in Adversity: Yeasts in the Agave Fermentation Environment

**DOI:** 10.1002/yea.3989

**Published:** 2025-02-19

**Authors:** Maritrini Colón‐González, Xitlali Aguirre‐Dugua, Mariana G. Guerrero‐Osornio, J. Abraham Avelar‐Rivas, Alexander DeLuna, Eugenio Mancera, Lucia Morales

**Affiliations:** ^1^ Laboratorio Internacional de Investigación sobre el Genoma Humano (LIIGH) Universidad Nacional Autónoma de México Querétaro México; ^2^ Unidad de Genómica Avanzada Centro de Investigación y de Estudios Avanzados del Instituto Politécnico Nacional Irapuato México; ^3^ Investigadoras e Investigadores por México, Consejo Nacional de Humanidades, Ciencias y Tecnologías (CONAHCYT) Ciudad de México México; ^4^ Instituto de Ecología Universidad Nacional Autónoma de México, Posgrado en Ciencias Biológicas Ciudad de México México; ^5^ Departamento de Ingeniería Genética Centro de Investigación y de Estudios Avanzados del Instituto Politécnico Nacional, Unidad Irapuato Irapuato México

**Keywords:** agave, domestication, fermentation, microbiome, yeast

## Abstract

Agave spirits have gained global recognition and hold a central position within the cultural heritage of Mexico. Traditional distilleries, characterized by open fermentations driven by local microbial communities, persist despite the rise of industrial‐scale counterparts. In this review, we explore the environmental conditions and production practices that make the must of cooked agave stems a unique habitat for colonizing microorganisms. Additionally, we review selected studies that have characterized yeast species within these communities, with a focus on their metabolic traits and genomic features. Over 50 fungal species, predominantly Saccharomycetales and few Basidiomycetes, along with a similar number of lactic and acetic acid bacteria, have been identified. Despite variations in the chemical composition of the agave substrates and diversity of cultural practices associated with each traditional fermentation process, yeast species such as *Saccharomyces cerevisiae, Kluyveromyces marxianus, Torulaspora delbrueckii*, and several *Pichia* species have been consistently isolated across all agave spirit‐producing regions. Importantly, cooked agave must is rich in fermentable sugars, yet it also contains inhibitory compounds that influence the proliferation dynamics of the microbial community. We discuss some of the genetic traits that may enable yeasts to flourish in this challenging environment and how human practices may shape microbial diversity by promoting the selection of microbes that are well‐adapted to agave fermentation environments. The increasing demand for agave spirits, combined with concerns about the preservation of natural resources and cultural practices associated with their production, underscores the need to deepen our understanding of all key players, including the yeast communities involved.

## Summary

- We outline the environmental context of yeast in open agave fermentations.
- We review the evolutionary history and adaptations of yeast in this habitat.
- We discuss how human practices affect microbial diversity in this environment.

## Graphical abstract

Three key players interacting in the agave fermentation environment: humans, agave plants, and microbes. Human selection of agaves with specific traits, combined with fermentation practices, may have created an environment that fosters the development of adaptive traits in certain microbial species, enabling them to thrive in the unique and challenging conditions of agave fermentation.

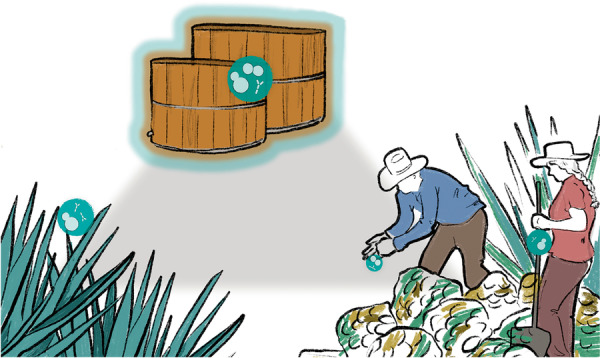

**Box**

**How Might the Selection of Microbial Communities Relate to Agave Domestication?**

Archaeological evidence indicates that the fermentation of cooked agave stems has been an established practice in Mesoamerica for over 3500 years (Bruman 2000; CONABIO 2006). Historically, this fermentation occurred seasonally, during the dry seasons whereby yeasts likely remained within or around the fermentation facilities, set to inoculate and rapidly proliferate in the next available batch of cooked agave. This cycle involved the movement of microorganisms between the fermentation tanks and the surrounding environment, aided by human activities and other animal vectors.

Three key players are involved in this intricate agave fermentation system: the agave plants, humans, and the fermenting microbial communities. The role of humans in the domestication of several agave species across the Americas is well documented. Since ancient times, human groups favored bigger, sweeter, and less harsh agave plants. These specific traits were intentionally selected for facilitating access and use by humans. It is also likely that another selected trait was the agaves' suitability for fermentation, enhancing the rapid proliferation of microorganisms capable of growing and fermenting its sugars. This factor likely influenced the domestication of species such as *Agave tequilana, A. rhodacantha*, *A. angustifolia*, and *A. karwinskii*, which are among the most strongly managed of the over 50 agave taxa used for spirit production. Concurrently, the microbial communities that efficiently convert sugars into ethanol, may also have been selected and co‐domesticated with the agaves, either through repitching practices or by reusing containers where successful fermentations had previously occurred.

Furthermore, the seasonal yet consistent availability of this human‐made substrate could unintentionally promote the proliferation of microbes adapted to this specific environment. This form of artificial selection might operate at both the community and genotype levels. At the community level, it potentially favors specific combinations of microbial species. At the genotype level, it would select genetic variants that enhance strain fitness in cooked agave must—traits such as resistance to ethanol and other growth inhibitors. These variants may also enhance survival under the typical intermittent conditions found in these facilities.

The distinct composition of yeast communities in agave fermentations, compared to surrounding natural habitats, suggests an active selection within this environment (Lachance 1995). Furthermore, at the yeast population level, the genomes of *S. cerevisiae* strains from agave fermentations reported to date are mostly grouped in a monophyletic Neotropical cluster and share traits associated with domestication. These traits include a high number of introgressed regions, numerous open reading frames (ORFs) with copy number variants, and elevated levels of average heterozygosity compared to wild strains (Avelar‐Rivas et al. 2024; Han et al. 2021; Peter et al. 2018; Pontes et al. 2020). These findings suggest that the close ecological relationship between agave related yeasts and humans may have shaped the observed yeast genetic diversity. Further research is essential for understanding their evolutionary dynamics.

**Which Traits in Yeast Isolates From Agave Fermentation Could be Adaptive?**

Fermentation of agave must expose microorganisms to fluctuating stress conditions that hinder their growth and impact their metabolism. These challenges include high sugar concentration that increases osmotic pressure, the accumulation of ethanol which impacts viability, and the presence of inhibitory compounds such as saponins, vanillin, and furans. Compared to strains isolated from grape fermentations, *S. cerevisiae* strains isolated from cooked agave fermentations show superior performance when agave must is used as the substrate. Agave strains exhibit enhanced sugar consumption rates, increased fermentation efficiency, and improved ethanol tolerance (Arrizon et al. 2006; De la Torre‐González et al. 2016; Fiore et al. 2005).

Particular physicochemical conditions can also be encountered by microorganisms in the agave must depending on the plant species used in each region. It has been shown that *S. cerevisiae* strains isolated from *A. tequilana* or *A. angustifolia* musts are unable to grow in musts from *A. salmiana* or *A. durangensis*, which contain higher concentrations of saponins (Alcazar‐Valle et al. 2019). If yeast populations are adapting to specific agave substrates, it is possible that the differential distribution of agave species across Mexico's territory may promote the divergence of associated yeast populations.

A comparative analysis between the microbial communities in agave fermentation and those found in other fermentative processes, like winemaking or beer‐brewing, will illuminate both the adaptive traits in yeast that are common to fermentation environments and those unique to agave must. Overall, there is still much to learn about the intricate interplay between agaves, humans, and microbial communities in agave spirits production, paving the way for future research in this field.

## 1 Agaves, Humans, and Microbes: A Long‐Lasting Tripartite Interaction in the Americas

Agaves are a group of succulents whose name derives from the Greek “*noble”* and Latin “*admirable”*, reflecting their remarkable adaptability to arid environments; they all belong to the genus *Agave* (Asparagaceae). Across millennia, native peoples from Mesoamerica and Aridoamerica have harnessed the rich diversity and abundance of agaves for a wide array of purposes (Colunga‐GarcíaMarín et al. 2017). The plant's leaves and stems were used for building, while fibers and thorns were used to elaborate textiles; flowers, raw sap, and cooked stems were consumed as food (Colunga‐GarcíaMarín and Zizumbo‐Villarreal 2007; Gentry 1982; MacNeish and Byers 1967). Cooking these plants for human consumption was a common practice long before the advent of agriculture and persists to this day. This practice allows the long‐chained sugars (fructans) in the stems and leaves to be broken down into edible mono and disaccharides, not only increasing their sweetness and reducing the intrinsic stringency, but also enabling yeasts and bacteria to ferment them.

All agave‐based alcoholic beverages we enjoy today rely on the process of fermentation, be it from the raw sap or the cooked agave must (Ramírez‐Guzmán et al. 2019). When the raw sap is fermented, the product is a beverage known as *pulque* which is directly consumed. When the cooked must is fermented and then distilled, the final product is an agave spirit. While historical evidence indicates that cooked agaves were milled and mixed with water to obtain fermented beverages centuries ago, it is not clear when distillation was first incorporated. The widespread practice of distillation most likely began with the arrival of the Spaniards and the subsequent introduction of Filipino and Arab stills by the sixteenth and seventeenth centuries (Bruman 2000; Colunga‐GarcíaMarín et al. 2017; Colunga‐GarcíaMarín and Zizumbo‐Villarreal 2007; Zizumbo‐Villarreal 1996). However, it has been proposed that distillation of fermented agave was already practiced by pre‐Hispanic cultures as early as 400 B.C. (Serra‐Puche and Lazcano‐Arce 2016).

**Figure 1:**
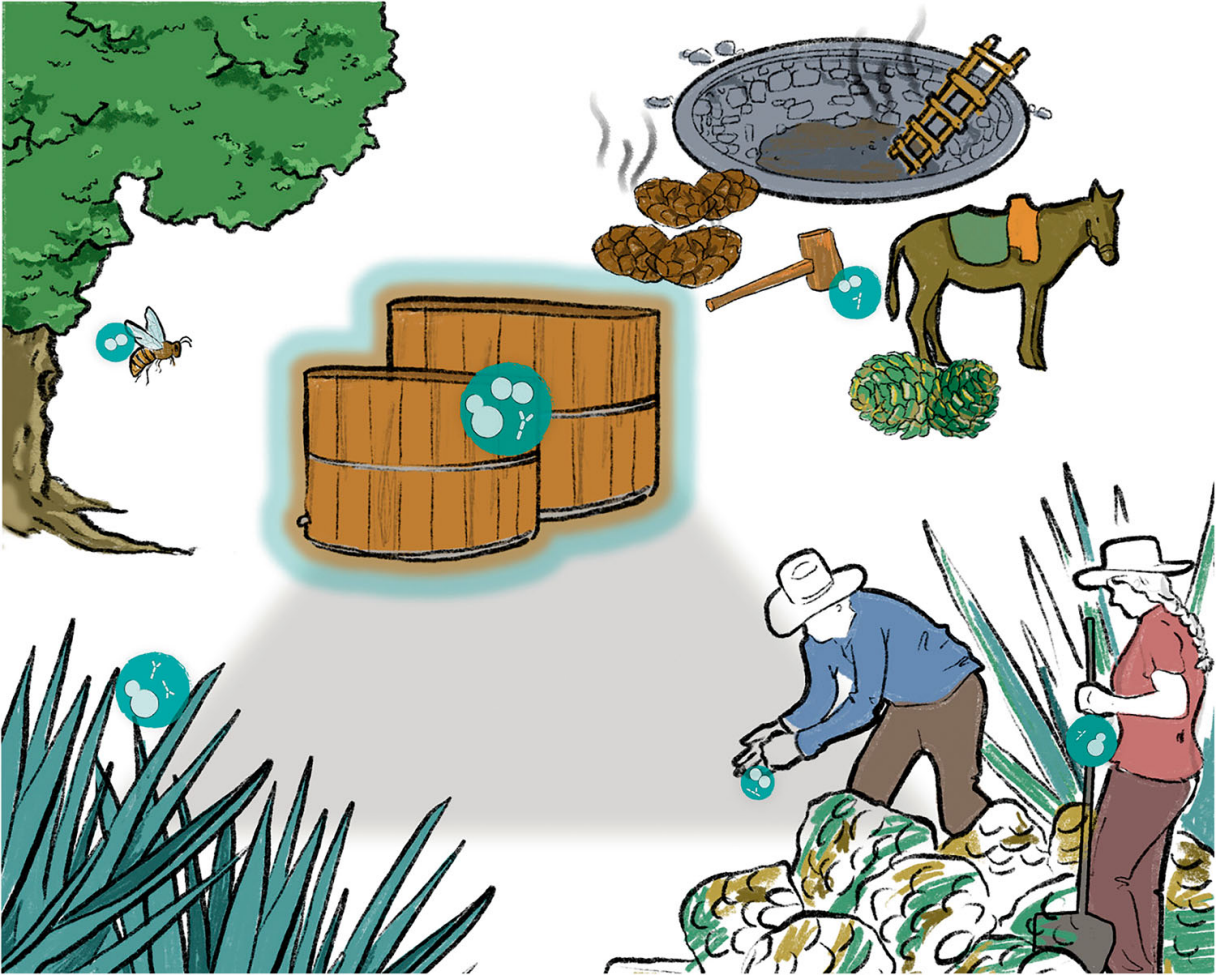
Representation of the interconnected elements that influence the agave fermentation microbial community. The three key players are connected by a triangle: agaves (left), producers who master the process (right), and open fermentation microbial communities (top). Humans have selectively chosen agaves with high sugar content and low saponin levels. Concurrently, they have selected microbial communities that are resilient to harsh compounds, capable of tolerating osmotic pressure, and efficient in producing high ethanol yields. Insects and other vectors facilitating the exchange of wild and human‐associated yeasts and bacteria between natural and human‐made settings are also shown.

The fermentation of cooked agave must to produce distillates emerged from the long‐lasting connection between agave plants, humans, and microorganisms. Producers have acquired empirical knowledge to master the fermentation process which inadvertently promotes the proliferation of adapted microorganisms. *Agave* species have undergone constant selection to increase their size and sugar content, and to reduce the production of toxic compounds such as saponins and oxalate crystals (Álvarez‐Ríos et al. 2020). For example, *A. tequilana*, frequently used in highly industrialized agave spirits production, shows a 5% to 32% reduction in saponin content compared to other agave species (Alcazar‐Valle et al. 2019). Domesticated variants of *A. salmiana* also exhibit larger sizes than the wild or less managed variants (Mora‐López et al. 2011). However, a critical factor in agave domestication was probably the selective pressure for must properties that could facilitate fermentation by microbial communities (Colunga‐GarcíaMarín et al. 2017). These communities may disperse back into the surrounding environment via various vectors, facilitating the exchange of wild and human‐associated yeasts and bacteria between natural and anthropogenic environments. This process potentially impacts microbial diversity in agave fermentations (Figure 1).

The standardization of the agave spirits production process, driven by an expanding market, as witnessed in tequila industry, incorporated the use of axenic inoculums for fermentation, which may cause a decline in the diversity of the microbial communities. The current review focuses on traditional open fermentations of cooked agave must as an ecological habitat for yeasts interacting with a varied array of other microbes.

Mexico is the primary producer of agave spirits. Since ancient times, a diverse array of cultural practices, combined with locally available natural resources, has been employed to craft various types of agave spirits (Zizumbo‐Villarreal et al. 2009). Agave harvesting, cooking, milling, fermentation, and distillation constitute the five main general steps used in Mexico's seven spirits producing regions. Agaves are harvested as soon as they are ready to reproduce sexually, since at this stage there is an accumulation of carbohydrates in the stem that would be used to develop the flower stalk. The nonreproductive stage varies among species, from 5 to 15 years (Arellano‐Plaza et al. 2022). Once the agave plants reach maturity, the leaves are trimmed off and the stem or heart (commonly known as *piña*) is cooked in either masonry ovens or earth pits. Cooking facilitates milling since it softens the stems and catalyzes the hydrolysis of fructans into fermentable sugars (Mancilla‐Margalli and López 2006; Waleckx et al. 2008). Milling extracts the juice and frees the sugars trapped in the fibers. This process is carried out using wooden mallets, stone mills (also named *tahonas*), press machines. The fermentation must is obtained from the juice of cooked and crushed agave with the addition of water. The must is then transferred to different types of containers, mainly tanks made of wood, masonry, plastic, or stainless steel. Occasionally, rarer containers such as stone hollows or hide sacks are also employed. Then, the fermentation step which will be discussed in the following sections, starts. Finally, the fermented agave must is distilled once or, more commonly, twice, and the alcoholic content is adjusted with water.

Since most small‐scale production settings lack the equipment to control the physicochemical variables of the process, producing a high‐quality artisanal spirit relies on the empirical knowledge and experience of the producers. This expertise masters how to deal with various factors influencing the aroma of agave spirits, such as agave species, plant age, cooking conditions and fermentation processes (Cedeño‐Cruz 2003; León‐Rodríguez et al. 2008; Pinal et al. 2009; Vera‐Guzmán et al. 2018; Vera‐Guzmán, Guzmán‐Gerónimo, and López 2010; Vera‐Guzmán, López, and Chávez‐Servia 2012). The organoleptic profile of agave spirits is attributed to alcohols (40%–80%), esters (8%–40%), acids (3%–26%), and acetals (1%–35%), with furans, terpenoids, ketones, phenols, and aldehydes making up the remaining 3%–7% (Molina‐Guerrero et al. 2007). Furans, pyrans, sulfur compounds, and ketones arise from Maillard reactions during cooking and fermentation, while compounds such as furfural, HMF, and vanillin result from thermal degradation of other plant compounds (Mancilla‐Margalli and López 2002). After distillation, some producers implement an aging or maturation step, which can significantly alter the concentration of volatile compounds in agave spirits (Acosta‐García et al. 2023; Cedeño‐Cruz 2003; López‐Ramírez et al. 2013; Mancilla‐Margalli and López 2002).

**Figure 2:**
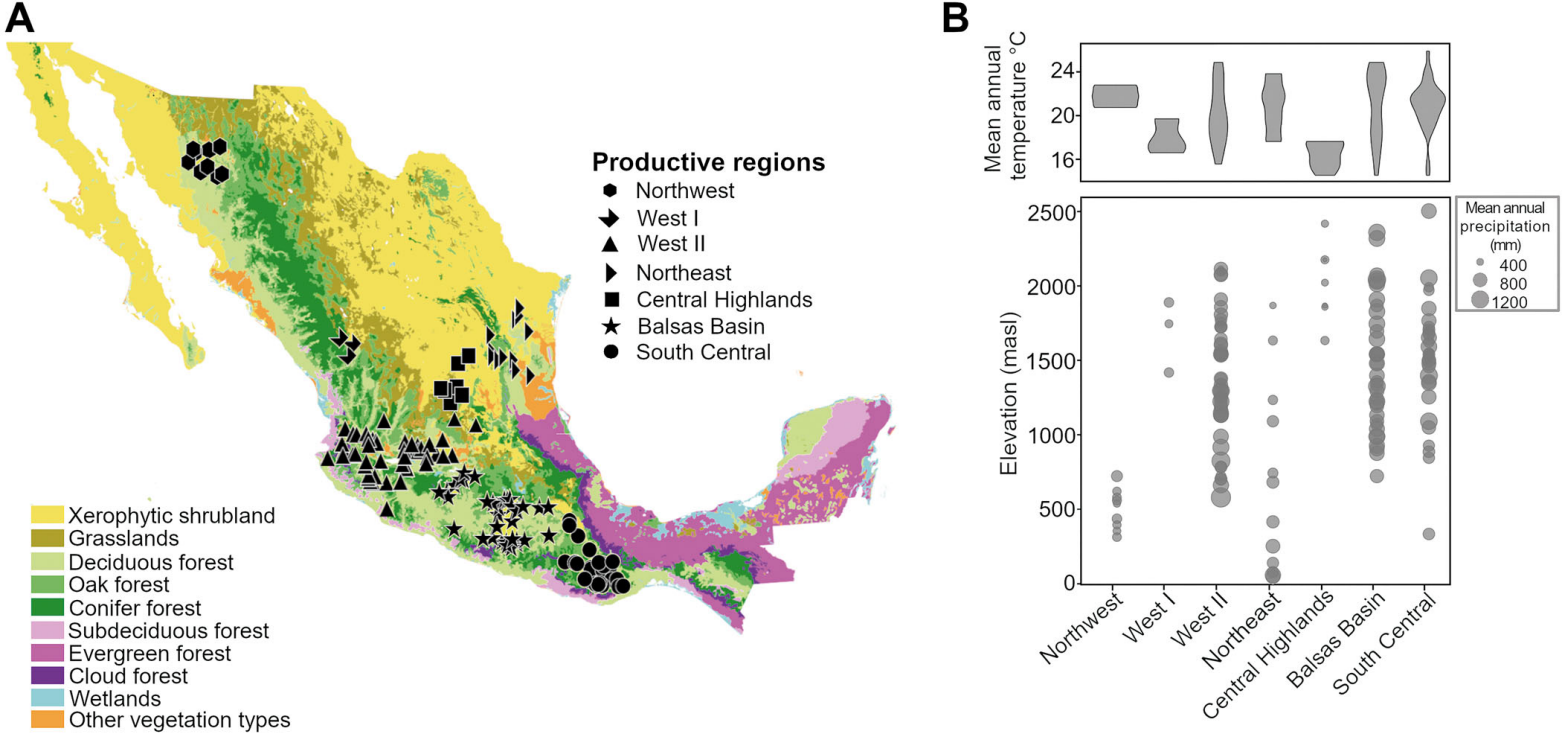
Geographic and climatic characteristics of traditional agave spirit factories. (A) Geographical distribution of representative traditional agave spirit‐producing municipalities in Mexico. The map is color‐coded based on vegetation types (INEGI 2003). Black symbols denote the location of these municipalities, with each figure corresponding to a distinct producing region (Aguirre, Illsley, and Larson 2006; Gallegos‐Casillas et al. 2024). (B) Climatic and environmental variables of municipalities, grouped by region. Upper panel: Violin plots depict the mean annual temperature of municipalities within each region. Lower panel: Elevation distribution (meters above sea level, masl) in municipalities within each producing region. The diameter of each circle represents the mean annual precipitation.

Agave spirits are produced in various types of ecosystems ranging from oak and deciduous forests to xerophytic shrublands (Figure 2A). The producing area extends from the northern border of Mexico with the USA to the southern states of Mexico, between 30°N and 16°S. This wide range encompasses areas with average annual rainfall ranging from 400 to 1500 mm, average annual temperatures between 15°C and 25°C, and elevations from 110 to over 2000 meters above sea level (Figure 2B).

There are seven regions where agave spirits are produced in Mexico: Northwest, Northeast, West I, West II, Balsas Basin, Central Highlands, and South Central (Aguirre, Illsley, and Larson 2006; Gallegos‐Casillas et al. 2024). The combination of geoclimatic characteristics such as temperature, precipitation, and elevation, are different in every producing region, and have therefore the potential to contribute to the uniqueness of the fermentation environment. In addition, Mexico is the center of diversity of the *Agave* genus. Out of the world's 200 agave species, 160 are found in Mexico (CONABIO 2009; García Mendoza 2002). Nowadays, agave spirits are produced from more than 50 *Agave* taxa, some more domesticated than others (Álvarez‐Ainza et al. 2017; Colunga‐GarcíaMarín et al. 2017; Gschaedler et al. 2017; Mora‐López et al. 2011). The selected species of agave, along with other factors, determine the four major types of agave spirits with Designation of Origin (DO) norms: *bacanora*, *tequila*, *raicilla*, and *mezcal*. Each type is defined by the following specific norms:

i. *Bacanora* is obtained specifically from *Agave angustifolia*, it can only be produced in the Northwestern state of Sonora (Gutiérrez‐Coronado, Acedo‐Félix, and Valenzuela‐Quintanar 2007; Ramírez‐Guzmán et al. 2019).
ii. *Tequila* relies on the blue variety of *A. tequilana*. *Tequila* can be produced in regions located in the states of Jalisco, Nayarit, Guanajuato, and Michoacan, in the Western region of Mexico, and Tamaulipas, in the Northeastern border of the country (Ramírez‐Guzmán et al. 2019).
iii. *Raicilla* is elaborated with *Agave maximiliana, A. inaequidens, A. valenciana, A. rhodacantha*, and *A. angustifolia*, among other agave species. *Raicilla* is produced in a small region located in the western states of Jalisco and Nayarit.
iv. *Mezcal*, its name derives from the Nahuatl words *metl* meaning “*maguey*” or agave, and *ixcalli* meaning “to roast”, thus *mezcal* means “roasted agave”. In contrast to *bacanora* and *tequila*, several agave species are used, among them *A. durangensis*, *A. americana*, *A. salmiana*, *A. maximiliana*, *A. rhodacantha*, *A. angustifolia, A. cupreata*, *A. potatorum*, *A. marmorata*, *A. karwinskii*, and *A. convallis*. The DO protection extends across the states of Durango, Zacatecas, San Luis Potosí, Guerrero, and Oaxaca, as well as some municipalities in the states of Tamaulipas, Guanajuato, Puebla, Morelos, Estado de México, Michoacán, Sinaloa and Aguascalientes (Cabrera‐Toledo et al. 2020; Colunga‐GarcíaMarín and Zizumbo‐Villarreal 2007; CONABIO 2006, 2009; Espinosa Paz et al. 2005; Jacques‐Hernandez, Herrera‐Perez, and Ramírez de León 2007; Tello‐Balderas and García‐Moya 2017; Vargas‐Ponce et al. 2009; Vázquez‐Pérez 2015).

While Designation of Origin (DO) norms set production guidelines and restrict the crafting of agave spirits to specific geographical areas and agave varieties, in some regions, there is still an artisanal production of agave spirits. These are often generically referred to as agave spirits, *mezcal*, or simply “*vino*” (*wine* in Spanish) and do not adhere to any official standards. Ultimately, the distinctiveness of each agave fermentation environment arises from a unique combination of agave species, climate, geography, microbial community and production practices, all contributing to the spirit's *terroir*.

### 1.1 The Chemical Properties of the Agave Must, the Fermentation Substrate

The chemical composition of the agave fermentation substrate, known as “must” is influenced by the agave species and their processing techniques. Must is a brown acidic liquid consisting of hydrolyzed agave juice and water, with varying amounts of bagasse. The reported pH of hydrolyzed agave must ranges from 4.0 to 4.8 (Sanchez‐Marroquin and Hope 1953; Waleckx et al. 2008). The must is a rich carbon source, with sugar concentrations ranging from 14 to 30 °Brix. Fructose, constituting up to 80% of the reducible sugar content in *A. tequilana* must, results from the thermal hydrolysis of the agave plant fructans, primarily agavins (Mancilla‐Margalli and López 2006; Sanchez‐Marroquin and Hope 1953; Waleckx et al. 2008). The must sugar content undergoes seasonal fluctuations; for example, in *A. angustifolia* must, the sugar concentration decreases from spring (295 g/L) to fall (170 g/L) (Vera‐Guzmán, López, and Chávez‐Servia 2012). This decrease is likely due to the dilution of sugar content resulting from increased water absorption during the rainy season.

Despite being a rich carbon source, cooked agave must is limited in nitrogen, with a total amino acid concentration close to 2.4 mg per liter, which is 130 times less than in raw agave sap (*aguamiel*), and around 1000 times less than in grape must (Díaz‐Montaño et al. 2008; Gutiérrez‐Gamboa et al. 2017; Ortiz‐Basurto et al. 2008; Sanchez‐Marroquin and Hope 1953; Valle‐Rodríguez et al. 2012; Waleckx et al. 2008). The reported carbon to nitrogen ratios fluctuate from 69 to 277 (Alcazar‐Valle et al. 2019) depending on the agave species (Hernández‐Cortés et al. 2016; Ortiz‐Basurto et al. 2008; Valle‐Rodríguez et al. 2012; Vera‐Guzmán, López, and Chávez‐Servia 2012).

Like carbon and nitrogen, the concentration of inhibitory compounds in the fermentation substrate, such as saponins, vanillin, and furans depends on the agave species and production practices. Saponins are plant constituents involved in the defense systems against insects and microbes. It has been reported that these compounds have inhibitory effects on yeast and bacterial growth, and in consequence, affect fermentation performance. Saponin concentrations in cooked agave vary from 293 to 431 ppm (Alcazar‐Valle et al. 2019). Compounds such as 5‐hydroxymethylfurfural (HMF), furfural, and vanillin are produced during the cooking step through the degradation of sugars and lignin (Cedeño‐Cruz 2003; Iwaki et al. 2013; Mancilla‐Margalli and López 2002; Molina‐Guerrero et al. 2007). At the end of the cooking process, their concentrations can reach up to 4000 ppm for HMF, 15 ppm for furfural, and 24 ppm for vanillin (Mancilla‐Margalli and López 2002). Additionally, other compounds present in agave must such as terpenes, aldehydes, furanones, ketones, pyrans, organic acids, and sulfur compounds are known to negatively affect microbial growth.

For fermentation to start, producers often dilute the cooked agave juice with water not only reducing the sugar concentration and the presence of inhibitory compounds, but also lowering osmotic pressure. In rare instances, though not widely accepted, additives such as urea, ammonium sulfate, ammonium phosphate, or magnesium sulfate are used to complement nitrogen deficiencies and prevent sluggish fermentation (Cedeño‐Cruz 2003).

There is a wide spectrum of practices related to the fermentation step in the agave spirit production. Traditionally, these fermentations are open and start without the deliberate introduction of an inoculum. However, due to the increasing demand for spirits, some distilleries inoculate with axenic starter cultures. Along this spectrum, various intermediate approaches also exist. Some producers accelerate the onset of fermentation by inoculating an entire community of microorganisms rather than a single isolated strain.

Non inoculated fermentation relays on microorganisms either carried over from the previous batches remaining in tanks or introduced from nearby reservoirs, such as distillery tools, facilities, or natural sources including animals, soil and vegetation. Oak trees (*Quercus spp*.), often found in forests near spirit production sites are known natural habitats for yeasts (Kowallik and Greig 2016; Sampaio and Gonçalves 2008; Spurley et al. 2021). In Mexico, these oak forests cover at least 4% of the territory and harbor over 30% of the world's oak species diversity (Valencia‐Avalos 2010). Insects such as fruit flies (*Drosophila*), beetles, bees and wasps may also transport yeast populations between fermentation tanks and their surroundings (Lachance 1995; Madden et al. 2018). Although few yeast*‐*insect associations have been described in this context, the majority of them involve *S. cerevisiae* and beetles, with *Drosophila* associations being less documented (Meriggi et al. 2020).

In some distilleries, a small fraction of a previous fermentation or *pulque* is used as an inoculum to initiate fermentation. *Pulque* is produced by fermenting raw agave sap, known as “*aguamiel*.” Since no cooking is involved in its preparation, the microbiome of the agave plant contributes to the fermentation. Although the microbiome associated with pulque fermentation and the agave plants falls outside the scope of this review, it is worth noting that this system has been extensively studied through both classical microbiology methods and metagenomic approaches (Álvarez‐Ríos, Figueredo‐Urbina, and Casas 2020; Astudillo‐Melgar et al. 2023; Chacón‐Vargas et al. 2020; Enríquez‐Salazar et al. 2017; Escalante et al. 2008, 2016, 2004; Lappe‐Oliveras et al. 2008; Ojeda‐Linares et al. 2021; Rocha‐Arriaga and Cruz‐Ramirez 2022; Rocha‐Arriaga et al. 2020).

At the other end of the spectrum, in more industrial settings, the need for larger and more uniform batches has led producers to inoculate agave fermentations with axenic yeast cultures. The market for yeast strains tailored to agave spirits offers fewer options compared to those for wine or beer. However, specific strains for tequila production are available, and many large spirit producers have developed their own strains. Interestingly, it has been suggested that mixed yeast cultures, rather than pure ones, improve the fermentation rate, ethanol yield, and aromatic profiles in agave spirit production, though further research is needed in this area (Acosta‐García et al. 2023; González‐Robles, Estarrón‐Espinosa, and Díaz‐Montaño 2015; Larralde‐Corona et al. 2021; Navarrete‐Bolaños and Serrato‐Joya 2023; Nuñez‐Guerrero et al. 2019). Based on self‐reported practices, the use of commercial inoculums remains uncommon in traditional distilleries.

### 1.2 The Microbiome of Agave Fermentations

Bacteria and microscopic fungi are among the most prevalent microorganisms in agave fermentations. Lactic acid bacteria (LAB), including *Lactobacillus brevis, L. casei*, *L. farraginis, L. kefir, L. plantarum* and *L. pontis, Weissella cibaria*, and *W. paramesenteroides*, contribute to the bacterial groups identified in these fermentations (Escalante‐Minakata et al. 2008; Kirchmayr et al. 2017). LAB fermentation of agave must leads to lactic acid accumulation, which acidifies the environment (Narváez‐Zapata et al. 2010) and may influence yeast proliferation (Escalante et al. 2016; Lappe‐Oliveras et al. 2008). Communities composed of yeasts and LAB are common in traditional alcoholic beverages fermented from maize, coconut, pineapple, or prickly pear in Mexico (Ojeda‐Linares et al. 2021), as well as in alcoholic beverages made from fermented rice, malt or apples such as sake, beer, and cider in other parts of the world (Bokulich et al. 2014; Tyakht et al. 2021). It has been suggested that the simultaneous presence of LAB and yeasts could stimulate metabolic changes in both microorganisms, thereby generating compounds that influence the organoleptic characteristics of the final product (De Vuyst and Leroy 2020; Narváez‐Zapata et al. 2010; Narvhus and Gadaga 2003).

Acetic acid bacteria (AAB) from the genera *Acetobacter* and *Gluconobacter* are part of another prominent bacterial group identified in agave fermentations (Escalante‐Minakata et al. 2008; Kirchmayr et al. 2017). The presence of AAB can influence the overall fermentation process by affecting the balance of microbial populations and contributing to the production of organic acids, which serve as substrates for the formation of certain volatile compounds. *Zymomonas mobilis*, known for its ability to produce ethanol is also prevalent in agave fermentations (Escalante‐Minakata et al. 2008; Kirchmayr et al. 2017). Spore‐forming bacteria have also been detected, their presence can be attributed to the contact between the cooked agave stems and either the soil or equipment used for milling (Kirchmayr et al. 2017).

Multiple studies have focused on identifying the yeast composition of the cooked‐agave microbiome. One of the first significant contributions was made over 25 years ago by Marc‐André Lachance, who conducted a comprehensive study of agave fermentation in a traditional *tequila* distillery using classical methods for yeast classification. Lachance sampled every stage of the *tequila* production process, from the harvesting of the agave plant to the fermentation itself, and even collected *Drosophila* specimens from the vicinity of the production site (Lachance 1995). This study concluded that the endogenous yeast species found in the agave plant, such as *Clavispora lusitaniae* and *Metschnikowia agaves* differ from those present in the fermentation tanks, where *S. cerevisiae*, *Maudiozyma humilis* and *Brettanomyces anomalus* were predominantly identified.

**Figure 3:**
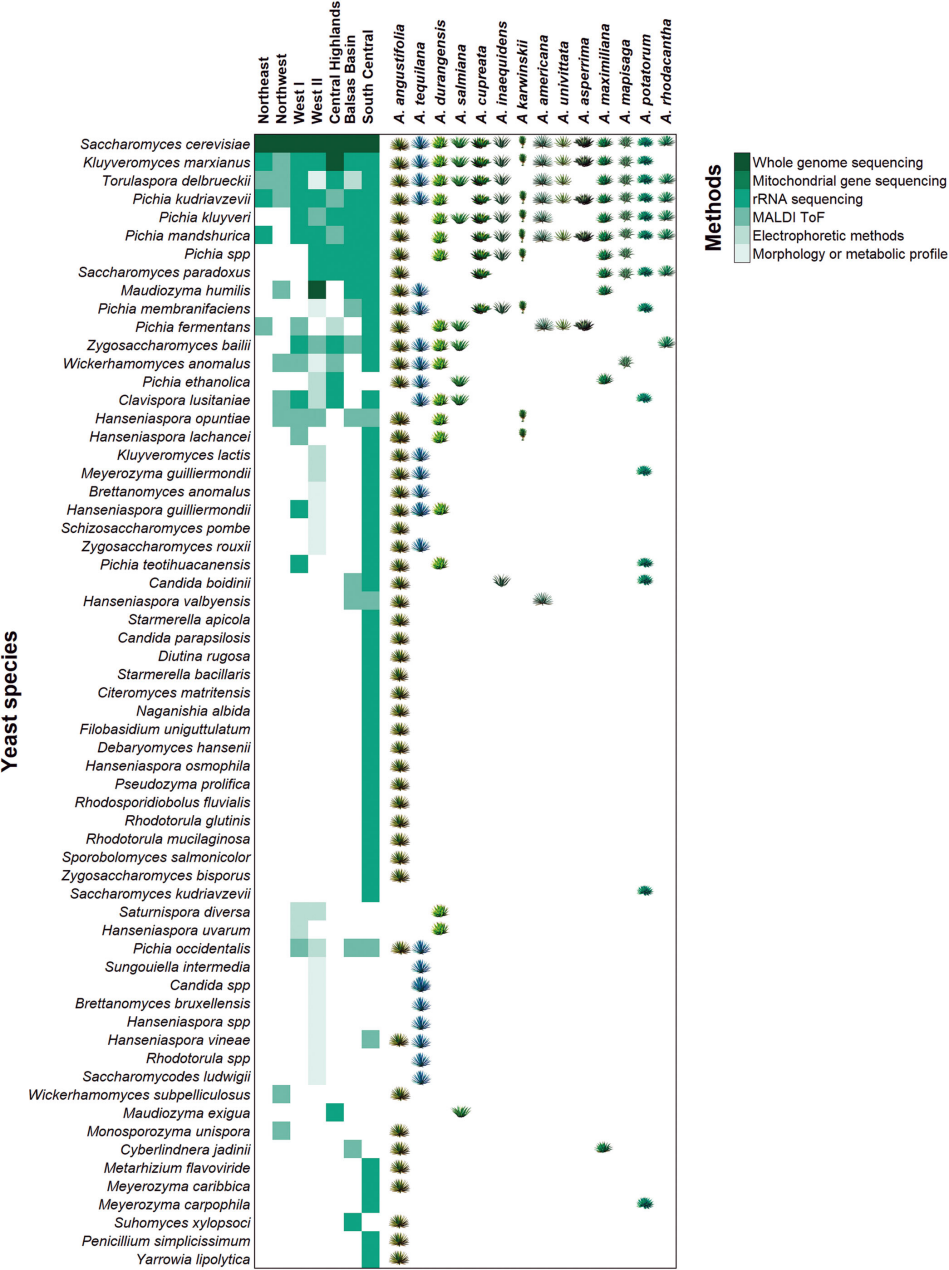
Yeast diversity in traditional agave fermentations in Mexico. To provide a comprehensive understanding of yeast diversity in this environment, we structured the data based on the seven agave spirit‐producing regions reported in (Gallegos‐Casillas et al. 2024). Left: The heatmap (green color scale) indicates the yeast identification method used; only the method with the highest precision for each species per region is shown. These methods include morphological and metabolic profiling, PCR‐RFLP, MALDI‐ToF, rRNA gene sequencing, mitochondrial gene sequencing, and whole genome sequencing. Right: Presence of yeast species in cooked agave must extracted from depicted agave species or mixtures containing such species. Agave illustrations are from Rafael Ruíz (CONABIO 2006).

Over 50 species of yeasts have been isolated from traditional agave fermentations coming from 15 different species of agave substrates. Figure 3 provides a comprehensive overview of the yeast species reported in this environment (Aldrete‐Tapia et al. 2018; Aldrete‐Tapia et al. 2020; Álvarez‐Ainza et al. 2015; Arias‐García 2008; Díaz‐Montaño et al. 2008; Escalante‐Minakata et al. 2008; Espinoza‐Martinez et al. 2023; Gallegos‐Casillas et al. 2024; García‐Ortega et al. 2022; Garibay‐Marcelo 2019; Gómez‐Márquez et al. 2022; Kirchmayr et al. 2017; Lachance 1995; Martínez‐Estrada et al. 2024; Nolasco‐Cancino et al. 2018; Páez‐Lerma et al. 2013; Peris et al. 2023; Peter et al. 2018; Verdugo Valdez et al. 2011). In most agave fermentations, it is generally observed that non‐*Saccharomyces* yeasts like *T. delbrueckii, K. marxianus*, *P. kluyveri*, and *Hanseniaspora spp*. proliferate during the early stages. As fermentation progresses and the ethanol levels rise, these yeasts are overtaken by species with higher ethanol tolerance, such as *S. cerevisiae* (Garibay‐Marcelo 2019; Kirchmayr et al. 2017; Lachance 1995; Nolasco‐Cancino et al. 2018; Páez‐Lerma et al. 2013; Verdugo Valdez et al. 2011; Walker et al. 2019).

Recent comprehensive surveys of microbial diversity in traditional agave fermentations in distilleries across all seven agave spirits‐producing regions in Mexico reveal that fungal communities remain relatively consistent throughout the fermentation process (Gallegos‐Casillas et al. 2024; Jara‐Servin et al. 2025). These studies also identified a core group of fungal species that are prominent in these fermentations, with *S. cerevisiae*, *T. delbrueckii*, *K. marxianus*, and several species of the genera *Pichia, Zygosaccharomyces* and *Hanseniaspora* being the most abundant. Among these core species, three species of filamentous fungi, *Penicillium polonicum*, *Mycosphaerella tassiana* and *Aureobasidium pullulans*, were newly identified, as they had not been previously reported in this fermentation environment (Jara‐Servin et al. 2025). Less commonly isolated yeasts include species from genera *Candida, Kazachstania*, and *Rhodotorula* (Gallegos‐Casillas et al. 2024) (Figure 3). Jara‐Servin and colleagues also identified more than 200 fungal species that had not been previously associated to agave fermentation environments. Of these, 81% belong to the Ascomycota phylum, 17% to the Basidiomycota, and the remaining 2% consist of species from the Mortierellomycota and Mucoromycota phyla. This finding expands our understanding of the fungal diversity present in agave fermentation, underscoring the complex microbial ecosystems that contribute to the production of agave spirits (Jara‐Servin et al. 2025).

Given their pervasive presence in agave fermentations, we will focus on describing the known characteristics of *S. cerevisiae, K. marxianus, T. delbrueckii*, and *Pichia spp*. isolates from this environment.

#### 1.2.1 Saccharomyces cerevisiae

*S. cerevisiae* has been isolated during all stages of fermentation from all the agave species that have been studied (Figure 3) (Aldrete‐Tapia et al. 2018; Aldrete‐Tapia et al. 2020; Álvarez‐Ainza et al. 2015; Arias‐García 2008; Avelar‐Rivas et al. 2024; Díaz‐Montaño et al. 2008; Escalante‐Minakata et al. 2008; Espinoza‐Martinez et al. 2023; Gallegos‐Casillas et al. 2024; Garibay‐Marcelo 2019; Jara‐Servin et al. 2025; Kirchmayr et al. 2017; Lachance 1995; Martínez‐Estrada et al. 2024; Nolasco‐Cancino et al. 2018; Páez‐Lerma et al. 2013; Peris et al. 2023; Peter et al. 2018; Verdugo Valdez et al. 2011). It has been reported that *S. cerevisiae* strains exhibit high genomic diversity between distilleries within the same state, and even among fermentation tanks in the same distillery (Aldrete‐Tapia et al. 2018; Álvarez‐Ainza et al. 2015; Páez‐Lerma et al. 2013). Major genomic studies have analyzed over 200 *S. cerevisiae* strains from the seven recognized agave spirit producing regions in Mexico. Their findings indicate that most of *S. cerevisiae* strains from agave fermentations cluster with a distinct lineage sharing a common ancestor with other Neotropical strains, such as human gut‐associated strains from French Guiana and wild strains from Brazil and Ecuador (Avelar‐Rivas et al. 2024; Peter et al. 2018; Pontes et al. 2020). Avelar‐Rivas et al. also reported a minority of isolates from agave fermentations (*n* = 5) grouping with the North American oak clade, along with a migrant strain grouping with the Wine clade.

Before the existence of this important resource of over 200 *S. cerevisiae* genomes from agave fermentations, evidence from mitochondrial markers strongly suggested the existence of diverse populations across the country (Páez‐Lerma et al. 2013). The whole genome analysis (Avelar‐Rivas et al. 2024) confirmed the high genomic diversity of the Mexican Agave isolates by identifying a main structuring of *S. cerevisiae* strains in ten populations arranged in three different phylogenetic clades: Mexican Agave 1, Mexican Agave 2 and Tequila Distillery. Moreover, the differentiation between Mexican Agave 1 and 2 correlates with isolation due to the geographical barrier of the mountain chain known as Sierra Madre Oriental. Additionally, strains from the western side of the Sierra Madre Oriental have a North to South gradient of genomic diversity, being the strains isolated from lower latitudes more diverse (Avelar‐Rivas et al. 2024).

Mexican Agave 1 strains appear to exhibit signs of domestication, including numerous regions with loss‐of‐heterozygosity, an increased frequency of ORFs with copy number variants, high levels of heterozygosity across the genome and the presence of the homing endonuclease VDE in the *VMA1* gene (O'Donnell et al. 2023; Peter et al. 2018). This version of the *VMA1* gene is absent in wild populations of *S. cerevisiae*, but it is present in domesticated strains used in both solid or liquid‐state fermentation (Han et al. 2021). These strains also harbor an allele of the *RTM1* gene associated with sucrose utilization and resistance to inhibitory compounds found in molasses. Furthermore, they have lost functional membrane aquaporins, which is hypothesized to be a consequence of domestication related to transitioning to high‐sugar niches (Pontes et al. 2020).

Notably, Mexican Agave *S. cerevisiae* strains exhibit an unusually high proportion of genes from the sister species *S. paradoxus* deriving from several pulses of introgression from the American lineages (Avelar‐Rivas et al. 2024; Peter et al. 2018; Tellini et al. 2024). Those genes include the *S. paradoxus* alleles of *BIO1/BIO6* encoding enzymes involved in biotin synthesis (Pontes et al. 2020). Importantly, *S. paradoxus* has also been isolated in the fermentation must of seven different agave species across five producing regions in Mexico (Figure 3) (Gallegos‐Casillas et al. 2024). This suggests that *Saccharomyces* interspecies interactions are relevant within the agave fermentation environment.

Experimental data also revealed possible adaptive traits within the *S. cerevisiae* strains from the agave fermentation environment. Autochthonous strains of *S. cerevisiae*, isolated from agave spirit distilleries can grow at 42°C, giving them a possible advantage in agave fermentations where temperature is not controlled and can reach up to 40°C inside tanks (Ruiz‐Terán et al. 2019). Flocculation, which may involve morphological changes in cell walls, appears to contribute to this thermotolerance. Notably, the flocculation‐related genes *FLO1, FLO5*, and *FLO11* are overexpressed in a strain isolated from an agave fermentation compared to a commercial wine strain (Vergara‐Álvarez et al. 2019). Moreover, the genes *OLE1, OLE2, ERG1*, *ERG11*, and *ERG25*, involved in the metabolism of oleic acid and ergosterol showed differential expression in a *S. cerevisiae* strain isolated from a *tequila* fermentation compared to a laboratory strain (Ramirez‐Córdova et al. 2012). These genes may be associated to ethanol tolerance, since concentrations in agave fermentation typically range from 4% to 9% (Cedeño‐Cruz 2003). Lastly, an agave *S. cerevisiae* strain displayed improved fermentation performance on high‐sugar substrates, such as agave must, compared to strains from grape must (Arrizon et al. 2006). This suggests that *S. cerevisiae* strains from agave fermentation may, in fact, have adapted to this environment.

#### 1.2.2 Kluyveromyces marxianus

*K. marxianus* is an aerobic yeast that utilizes respiro‐fermentative metabolism as an energy source (Lane and Morrissey 2010). Unlike *S. cerevisiae*, some *K. marxianus* strains can use lactose or inulin as carbon sources. This yeast has been identified in traditional fermentation of must of thirteen agave species used in the production of *bacanora, mezcal*, and *tequila* across Mexico (Figure 3) (Aldrete‐Tapia et al. 2020; Arias‐García 2008; Escalante‐Minakata et al. 2008; Gallegos‐Casillas et al. 2024; Garibay‐Marcelo 2019; Gómez‐Márquez et al. 2022; Kirchmayr et al. 2017; Lachance 1995; Martínez‐Estrada et al. 2024; Nolasco‐Cancino et al. 2018; Páez‐Lerma et al. 2013; Verdugo Valdez et al. 2011).

Three genomic sequences of *K. marxianus* from agave fermentation isolates have been reported so far. The first one was obtained from South‐Africa (Schabort et al. 2016), while the other two originate from Mexico: one from the Central Highlands region and the other from fermented must of *A. fourcroydes* in the Yucatan peninsula in the South of the country (Gómez‐Márquez et al. 2022; Lappe‐Oliveras et al. 2023; Lozano‐Aguirre et al. 2024). Since the Yucatan region and South‐Africa historically have not been spirit‐producing areas, these strains were not considered in the inventory presented in Figure 3.

Phylogenetic analysis suggests that the South‐African strain forms a unique, separate lineage that is highly divergent from both non‐dairy (A‐haplotypes) and dairy (B‐haplotypes) strains (Ortiz‐Merino et al. 2018). Typically, dairy fermentation isolates are diploid or triploid, while non‐dairy fermentation strains are haploid, leading to the hypothesis that isolation source and ploidy are related (Ortiz‐Merino et al. 2018). However, the Mexican strain from the Central Highlands is a diploid, with both A‐ and B‐haplotypes (Gómez‐Márquez et al. 2022). To establish a precise phylogenetic relationship between dairy and agave strains, a detailed genomic analysis of additional isolates from agave fermentations will be required.

Some *K. marxianus* strains isolated from agave must exhibit traits suggestive of adaptation to this environment, including increased tolerance to ethanol and saponins, as well as enhanced fructan assimilation capacity (Alcázar‐Valle 2016). Despite reports of high ethanol sensitivity, *K. marxianus* often emerges as the predominant non‐*Saccharomyces* species in the late stages of agave fermentations (Alvarez‐Ainza et al. 2021; Lachance 1995; Verdugo Valdez et al. 2011). Its elevated saponin tolerance, compared to *S. cerevisiae*, is attributed to higher concentrations of 1,3 ß‐glucans and mannans in its cell wall, and the induction of saponin‐hydrolyzing enzymes (Alcázar‐Valle 2016; Alcazar‐Valle et al. 2019). Additionally, *K. marxianus* strains from agave fermentations show increased fructanase activity relative to other yeasts, potentially enhancing their ability to assimilate fructans from agave hydrolysates (Arrizon et al. 2012).

Due to the high phenotypic variability among *K. marxianus* strains, determining whether the species has superior fermentative capacity compared to *S. cerevisiae* has been challenging. Some studies indicate that *K. marxianus* strains achieve higher ethanol production in agave must fermentation compared to *S. cerevisiae* strains (96% vs 70%), however, these observations seem to be strain‐dependent (Adame‐Soto et al. 2023; Amaya‐Delgado et al. 2013; López‐Alvarez et al. 2012). Regardless of the strain, when compared to *S. cerevisiae, K. marxianus* tends to produce more volatile compounds, particularly esters and higher alcohols like isoprenol, 3‐methylpentanol, linalool, nerolidol and thymol (Amaya‐Delgado et al. 2013; López‐Alvarez et al. 2012; Segura‐García et al. 2015). Consistently, agave fermentation involving mix cultures of yeast, including *K. marxianus*, have demonstrated a broader profile of aromatic compounds (Navarrete‐Bolaños and Serrato‐Joya 2023).

#### 1.2.3 Torulaspora delbrueckii

*T. delbrueckii* has been isolated from a variety of substrates including fruits, insects, soils, plants, seawater, spoiled food, and agave must (Kurtzman 2011). The species has been documented in fermentations from twelve agave species across every producing region in Mexico (Espinoza‐Martinez et al. 2023; Gallegos‐Casillas et al. 2024; Garibay‐Marcelo 2019; Kirchmayr et al. 2017; Lachance 1995; Martínez‐Estrada et al. 2024; Páez‐Lerma et al. 2013; Verdugo Valdez et al. 2011). In *A. salmiana* must, this yeast is present during both early and final stages of fermentation (Alvarez‐Ainza et al. 2021; Lachance 1995; Verdugo Valdez et al. 2011) and its occurrence in some distilleries may fluctuate in response to temperature (Martínez‐Estrada et al. 2024).

Unlike *S. cerevisiae, T. delbrueckii* can maintain a respiratory metabolism under low oxygen conditions, which likely influences its fermentative capacity (Fernandes et al. 2021). However, as with other yeasts, there is significant variability in the fermentative capacity and ethanol tolerance among strains (Fernandes et al. 2021; Kurtzman 2011; Nuñez‐Guerrero et al. 2016).

Similar to other non‐Saccharomyces yeasts, *T. delbrueckii* may play a role in enhancing the aroma and flavor profile of alcoholic beverages through the production of volatile compounds. Its contributions have been extensively studied in beer and wine, where it is associated with increased production of higher alcohols (Canonico, Comitini, and Ciani 2017; Tufariello et al. 2021). Agave spirits produced with mixed cultures of *S. cerevisiae* and *T. delbrueckii* show enrichment in esters and terpenes impacting the sensory properties of spirits (Nuñez‐Guerrero et al. 2016).

#### 1.2.4 Pichia Spp

*Pichia* species are predominant yeasts in the fermentation of sour milk, meat, acid curd cheese, olive, cacao, and coffee (Tofalo et al. 2020). They inhabit diverse natural environments including fruits, rotted fruits, plant tissues, human sputum, and animals. Several species from this genus, have been isolated from fermentations of 14 distinct agave species across all producing regions, being the most prevalent *P. kudriavzevii, P. kluyveri*, and *P. manshurica* (Figure 3) (Aldrete‐Tapia et al. 2020; Arias‐García 2008; Escalante‐Minakata et al. 2008; Gallegos‐Casillas et al. 2024; Garibay‐Marcelo 2019; Kirchmayr et al. 2017; Lachance 1995; Nolasco‐Cancino et al. 2018; Páez‐Lerma et al. 2013; Verdugo Valdez et al. 2011). *P. kudriavzevii* and *P. manshurica* are more prevalent in early stages of agave spirit fermentation (Nolasco‐Cancino et al. 2018), while *P. membranifaciens* has been found in all stages of *tequila* fermentation (Lachance 1995). The presence of *P. kluyveri* is correlated with changes in temperature (Martínez‐Estrada et al. 2024). Importantly, two candidate new species of *Pichia* isolated from agave fermentations were reported by Gallegos‐Casillas et al. (2024); one of these species was recently described formally as *P. teotihuacanensis* (Chai et al. 2024).

There is growing interest in understanding the mechanisms that enhance ethanol yield and flavor profiles in mixed yeast cultures involving *Pichia* species. For example, sugar consumption by *P. kudriavzevii* was two‐fold higher when co‐cultivated with *S. cerevisiae* or *K. marxianus* than in pure cultures, potentially increasing ethanol production. Additionally, the presence of *P. kudriavzevii* has been linked to increased concentrations of higher alcohols, which can either positively or negatively impact the aroma and flavor (Liu et al. 2016; Nolasco‐Cancino et al. 2018). During *tequila* fermentation, *P. kluyveri* produces a greater quantity of esters compared to *S. cerevisiae*, these compounds play a role in enhancing the fragrance of distilled spirits (Amaya‐Delgado et al. 2013; Méndez‐Zamora et al. 2021; Segura‐García et al. 2015). Overall, these findings highlight the contribution of non‐*Saccharomyces* yeasts to the flavor and aroma of agave spirits.

### 1.3 Other Species

Apart from the species described above, members of the *Hanseniaspora* genus have attracted attention due to their ability to synthesize a variety of aromatic compounds. Commonly found in fruits, flowers, and tree bark, several *Hanseniaspora* species have been identified in the fermentations of *A. angustifolia*, *A. durangensis*, *A. tequilana*, *A. karwinskii*, and *A. americana* musts (Figure 3) across the Northwest, West I, West II, Balsas Basin, and South Central producing regions (Arias‐García 2008; Díaz‐Montaño et al. 2008; Gallegos‐Casillas et al. 2024; Kirchmayr et al. 2017; Lachance 1995; Martínez‐Estrada et al. 2024; Páez‐Lerma et al. 2013).

*Maudiozyma* and *Monosporozyma* species have been identified in Northwest, West II, Balsas Basin, Central Highlands, and South Central producing regions, in fermentations of *A. angustifolia, A. maximiliana, A. tequilana*, and *A. salmiana* (Gallegos‐Casillas et al. 2024; García‐Ortega et al. 2022; Lachance 1995; Verdugo Valdez et al. 2011). Genomic analyzes of *Maudiozyma humilis* (previously reported as *Kazachstania humilis*) from agave revealed that these strains cluster together and are divergent from other sourdough strains. Compared to the reference strain, agave isolates of *M. humilis* exhibit relatively low genetic identity, suggesting that they form a distinct population. This divergence is further supported by rearrangements, segmental and dispersed duplications and other structural variants, which likely arise from adaptation to their specific niche. For instance, a duplication of the gene *ZWF1*, associated with furan tolerance in *S. cerevisiae*, was identified in an agave‐derived *M. humilis* strain (Gallegos‐Casillas et al. 2024).

In summary, various yeast species are commonly found in agave fermentations, and when their genomes have been analyzed they formed distinct phylogenetic groups that separate them from strains isolated from wine, beer, or other fermentations (Avelar‐Rivas et al. 2024; Gallegos‐Casillas et al. 2024; Páez‐Lerma et al. 2013). However, the diverse chemical composition of fermenting musts implies that adaptive traits may differ, even among isolates from the same species. This leads to metabolic diversity within these specialized microbial communities. Further research is crucial to understand the specific contributions of each strain and species together with their interactions.

## 2 Conclusions and Perspectives

Agave spirits, deeply rooted in Mexican culture, have gained global appreciation for the richness of their crafting processes and their distinctive organoleptic profiles. These spirits, primarily obtained through non‐inoculated fermentation, depend on a microbial consortium of yeasts and bacteria that thrive at the interface of natural and human environments. This practice of non‐inoculated fermentation of cooked agave dating back to pre‐Hispanic cultures suggests that microbial communities in this habitat have evolved specific traits to thrive in high sugar environments with harsh inhibitory compounds. Despite this long history, the extent to which the microbial community and their specific enzymatic activities contribute to the agave spirits' bouquet is yet to be fully understood. The yeast pathways involved in the production of organoleptic compounds have been extensively reviewed by Dzialo and colleagues (Dzialo et al. 2017). However, other factors such as the interaction between different microbial species and the interplay between the chemical properties of the substrates and the microorganisms, may also play a crucial role in determining the final sensory profile of agave spirits. Systematic studies comparing the organoleptic profiles of agave fermentations using the same microbial communities grown on different agave substrates would shed light on how the chemical composition of the agave must influences flavor and aroma. Conversely, examining the sensory outputs produced by different microbial communities fermenting the same agave substrate would further elucidate the complex role of microbial interactions and their relative contributions to aroma and flavor.

Agave fermentations occur in diverse ecosystems, and due to their artisanal nature, each distillery employs a unique combination of practices across the five steps required to produce spirits. These factors influence the fungal microbial communities of agave fermentations which appear to be primarily determined by the local characteristics and practices of each production site; the distillery itself plays a significant role in shaping the diversity of both bacterial and fungal communities (Jara‐Servin et al. 2025). However, these studies have mainly focused on diversity at the species level, and it remains to be determined whether the observed intraspecific fungal diversity is correlated with geographical distribution.

Deep sampling and sequencing efforts targeting yeasts from agave fermentations have enriched our understanding of their ecological origins, phylogenetic relationships, and evolutionary dynamics, particularly for the model species, *S. cerevisiae*. These studies reveal significant diversity and multiple introgression events from its sister species, *S. paradoxus*. However, similar studies focusing on other *Saccharomyces* and non‐*Saccharomyces* yeast species, which also play crucial roles in fermenting cooked agave, remain scarce.

Increasing global demand poses a threat to traditional agave spirit producing methods and, consequently, to the diversity of both the agave plants and associated microorganisms. Research into the genomic and phenotypic characteristics of the entire yeast community from agave fermentations will deepen our understanding of microbial dynamics and evolution. This knowledge could be applied to enhance the efficiency and sustainability of industrial production while preserving the natural resources involved.

## Author Contributions

Maritrini Colón‐González and Lucia Morales conducted the primary literature search and data compilation. Alexander DeLuna, Eugenio Mancera, Xitlali Aguirre‐Dugua, Mariana G. Guerrero‐Osornio, J. Abraham Avelar‐Rivas assisted with additional searches and data analysis. Maritrini Colón‐González and Lucia Morales drafted the manuscript while all authors contributed to writing specific sections and creating the figures. All authors participated in revising the final draft.

## Conflicts of Interest

The authors declare no conflicts of interest.

## Supporting information

Supporting information

## Data Availability

The authors have nothing to report.
