# Supplementary material for: Thriving in Adversity: Yeasts in the Agave Fermentation Environment
Source: Yeast. 2025 Feb 19;42(1-3):16–30. doi: 10.1002/yea.3989 (PMC11891984; doi:10.1002/yea.3989)
Supplement: Supplementary file 1 — Supporting information [file YEA-42-16-s001.pdf]

Thriving in Adversity: Yeasts in the Agave Fermentation Environment

Prosperando en la Adversidad: Levaduras en el Ambiente de Fermentación del Agave

Maritrini Colón-González[1,2], Xitlali Aguirre-Dugua[3], Mariana G. Guerrero-Osornio[1,4], J. Abraham Avelar-Rivas[2], Alexander DeLuna[2], Eugenio Mancera[5], Lucía Morales[1]

[1] *Laboratorio Internacional de Investigación sobre el Genoma Humano (LIIGH), Universidad Nacional Autónoma de México, Querétaro, México*

[2] *Unidad de Genómica Avanzada, Centro de Investigación y de Estudios Avanzados del Instituto Politécnico Nacional, Irapuato, México*

[3] *Investigadoras e Investigadores por México, Consejo Nacional de Humanidades, Ciencias y Tecnologías (CONAHCYT), Ciudad de México, México*

[4] *Instituto de Ecología, Universidad Nacional Autónoma de México, Posgrado en Ciencias Biológicas, Ciudad de México, México*

[5] *Departamento de Ingeniería Genética, Centro de Investigación y de Estudios Avanzados del Instituto Politécnico Nacional, Unidad Irapuato, Irapuato, México*

* Correspondencia a: Lucia Morales (lmorales@liigh.unam.mx)

Palabras clave: agave, domesticación, fermentación, levadura, microbioma

Título corto: Levaduras en las fermentaciones de agave

Mensaje principal:

• Describimos el contexto ambiental de las levaduras en las fermentaciones abiertas de agave.

• Revisamos la historia evolutiva y las adaptaciones de las levaduras en este hábitat.

• Discutimos cómo las prácticas humanas afectan la diversidad microbiana en este entorno.

RESUMEN

Los destilados de agave han ganado reconocimiento mundial y ocupan un lugar fundamental en el patrimonio cultural de México. Las destilerías tradicionales, donde las que las fermentaciones son abiertas y no inoculadas, permitiendo que el jugo de agave sea colonizado por comunidades microbianas locales, siguen siendo una parte esencial en la producción de estos destilados, a pesar del auge de las destilerías a gran escala. En esta revisión, analizamos las condiciones ambientales y las prácticas de producción que convierten al mosto de agave en un hábitat único para los microorganismos colonizadores. Además, examinamos estudios seleccionados que han caracterizado las especies de levaduras dentro de estas comunidades, enfocándonos en sus características metabólicas y genómicas. Se han identificado más de cincuenta especies de hongos, en su mayoría del orden Saccharomycetales y algunas especies de Basidiomycetes, así como un número similar de bacterias lácticas y acéticas. A pesar de las variaciones en la composición química de los sustratos de agave y la diversidad de prácticas culturales asociadas con cada proceso de fermentación tradicional, especies de levaduras como *Saccharomyces cerevisiae*, *Kluyveromyces marxianus*, *Torulaspora delbrueckii* y varias especies de *Pichia* se han aislado de manera consistente en todas las regiones productoras de destilados de agave. Es importante destacar que el mosto de agave cocido es rico en azúcares fermentables, pero también contiene compuestos inhibitorios que afectan la proliferación de la comunidad microbiana. En este sentido, discutimos algunos de los rasgos genéticos que podrían permitir que las levaduras prosperen en este entorno tan desafiante, así como la forma en que las prácticas humanas pueden moldear la diversidad microbiana, promoviendo la selección de microorganismos que están mejor adaptados a los ambientes de fermentación del agave. La creciente demanda de los destilados de agave, sumada a las preocupaciones sobre la conservación de los recursos naturales y las prácticas culturales asociadas con su producción, subraya la necesidad de profundizar en nuestro entendimiento de todos los elementos clave en este proceso, incluidas las comunidades de levaduras involucradas.

1. PREGUNTAS A DISCUSIÓN

1.1. ¿Cómo podría la selección de comunidades microbianas relacionarse con la domesticación del agave?

La evidencia arqueológica indica que la fermentación del mosto de agave ha sido una práctica establecida en Mesoamérica durante más de 3,500 años (Bruman, 2000; CONABIO, 2006). Históricamente, esta fermentación ocurría de manera estacional, durante las temporadas secas, en las que las levaduras probablemente permanecían dentro o alrededor de las instalaciones de fermentación, listas para inocularse y proliferar rápidamente en el siguiente lote disponible de agave cocido. Este ciclo involucraba el movimiento de microorganismos entre los tanques de fermentación y el entorno circundante, facilitado por actividades humanas y otros vectores animales.

Tres actores clave están involucrados en este complejo sistema de fermentación del agave: las plantas de agave, los seres humanos y las comunidades microbianas fermentadoras. El papel de los humanos en la domesticación de varias especies de agave a través de América está bien documentado. Desde tiempos antiguos, los grupos humanos favorecieron a las plantas de agave más grandes, dulces y menos ásperas. Estas características específicas fueron seleccionadas intencionalmente para facilitar su acceso y uso por parte de los seres humanos. Es probable que otra característica seleccionada fuera la idoneidad de los agaves para la fermentación, lo que mejoraba la rápida proliferación de microorganismos capaces de fermentar sus azúcares. Este factor probablemente influyó en la domesticación de especies como *Agave tequilana*, *A. rhodacantha*, *A. angustifolia* y *A. karwinskii*, que se encuentran entre las más utilizadas de los más de 50 taxones de agave utilizados para la producción de bebidas alcohólicas. Al mismo tiempo, las comunidades microbianas que convierten eficientemente los azúcares en etanol también pudieron haber sido seleccionadas y co-domesticadas junto con los agaves, ya sea a través de prácticas de reutilización de cultivos o mediante el reciclaje de recipientes en los que previamente habían ocurrido fermentaciones exitosas.

Además, la disponibilidad estacional pero constante de este sustrato creado por el ser humano podría promover de manera no intencional la proliferación de microorganismos adaptados a este ambiente específico. Esta forma de selección artificial podría operar tanto a nivel comunitario

como genotípico. A nivel comunitario, potencialmente favorece combinaciones específicas de especies microbianas. A nivel genotípico, seleccionaría variantes genéticas que mejoran la aptitud de las cepas en el mosto de agave cocido—características como la resistencia al etanol y otros inhibidores del crecimiento. Estas variantes también podrían mejorar la supervivencia bajo las típicas condiciones intermitentes encontradas en estas instalaciones.

La composición distintiva de las comunidades de levaduras en las fermentaciones de agave, en comparación con los hábitats naturales circundantes, sugiere una selección activa dentro de este entorno (Lachance, 1995). Además, a nivel de la población de levaduras, los genomas de las cepas de *S. cerevisiae* provenientes de fermentaciones de agave reportadas hasta la fecha se agrupan principalmente en un clúster monofilético Neotropical y comparten características asociadas con la domesticación. Estas características incluyen un alto número de regiones introgresadas, numerosos marcos de lectura abiertos (ORFs) con variantes en el número de copias, y niveles elevados de heterocigosidad promedio en comparación con las cepas silvestres (Avelar-Rivas et al., 2024; Han et al., 2021; Peter et al., 2018; Pontes et al., 2020). Estos hallazgos sugieren que la estrecha relación ecológica entre las levaduras relacionadas con el agave y los humanos pudo haber modelado la diversidad genética observada en las levaduras. Se requiere más investigación para comprender sus dinámicas evolutivas.

1.2. ¿Qué características de los aislamientos de levaduras provenientes de la fermentación de agave podrían ser adaptativas?

La fermentación del mosto de agave expone a los microorganismos a condiciones de estrés fluctuantes que obstaculizan su crecimiento e impactan su metabolismo. Estos desafíos incluyen altas concentraciones de azúcares que aumentan la presión osmótica, la acumulación de etanol que afecta la viabilidad, y la presencia de compuestos inhibitorios como saponinas, vainillina y furanos. En comparación con las cepas aisladas de fermentaciones de uva, las cepas de *S. cerevisiae* aisladas de fermentaciones de agave cocido muestran un mejor desempeño cuando se utiliza el mosto de agave como sustrato. Las cepas de agave exhiben tasas mejoradas de consumo de azúcares, mayor eficiencia en la fermentación y mejor tolerancia al etanol (Arrizon et al., 2006; De la Torre-González et al., 2016; Fiore et al., 2005).

También pueden encontrarse condiciones fisicoquímicas particulares que los microorganismos deben enfrentar en el mosto de agave, dependiendo de la especie de planta utilizada en cada

región. Se ha demostrado que las cepas de *S. cerevisiae* aisladas de mostos de *A. tequilana* o *A. angustifolia* no pueden crecer en mostos de *A. salmiana* o *A. durangensis*, que contienen concentraciones más altas de saponinas (Alcazar-Valle et al., 2019). Si las poblaciones de levaduras se están adaptando a sustratos específicos de agave, es posible que la distribución diferencial de las especies de agave a través del territorio mexicano favorezca la divergencia de las poblaciones de levaduras asociadas.

Un análisis comparativo entre las comunidades microbianas en la fermentación de agave y aquellas encontradas en otros procesos fermentativos, como la vinificación o la elaboración de cerveza, ayudará a entender tanto las características adaptativas de las levaduras comunes en los entornos de fermentación como aquellas únicas del mosto de agave. En general, aún queda mucho por aprender sobre la compleja interacción entre los agaves, los seres humanos y las comunidades microbianas en la producción de bebidas alcohólicas de agave, lo que abre el camino para futuras investigaciones en este campo.

2. AGAVES, HUMANOS Y MICROORGANISMOS: UN TRIÁNGULO ANTIGUO EN LAS AMÉRICAS

Los agaves son un grupo de suculentas cuyo nombre proviene del griego “noble” y del latín “admirable”, lo que refleja su notable capacidad de adaptación a ambientes áridos; todos pertenecen al género *Agave* (Asparagaceae). A lo largo de milenios, los pueblos originarios de Mesoamérica y Aridoamérica han aprovechado la rica diversidad y abundancia de los agaves para una amplia variedad de propósitos (Colunga-GarcíaMarín et al., 2017). Las hojas y los tallos de la planta se utilizaban para la construcción, mientras que sus fibras y espinas se usaban para elaborar textiles; las flores, la savia cruda y las piñas (el corazón o parte central de la planta) cocidas eran consumidas como alimento (Colunga-GarcíaMarín y Zizumbo-Villarreal, 2007; Gentry, 1982; MacNeish y Byers, 1967). La práctica de cocinar estas plantas para el consumo humano fue común mucho antes del advenimiento de la agricultura y persiste hasta el día de hoy. Este proceso permite que los azúcares de cadena larga (fructanos) presentes en las piñas y las hojas se descompongan en monosacáridos y disacáridos comestibles, lo que no solo incrementa su dulzura y reduce la astringencia intrínseca, sino que también permite que levaduras y bacterias los fermenten.

Todas las bebidas alcohólicas basadas en agave que consumimos hoy en día dependen del proceso de fermentación, ya sea de la savia cruda o del mosto de agave cocido (Ramírez-Guzmán et al., 2019). Cuando la savia cruda se fermenta, el producto es una bebida conocida como pulque, que se consume directamente. Cuando el mosto cocido se fermenta y luego se destila, el producto final es un destilado de agave. Aunque las evidencias históricas indican que las piñas de agave cocidas se molían y se mezclaban con agua para obtener bebidas fermentadas desde hace siglos, no está claro cuándo se incorporó por primera vez la destilación. La práctica extendida de la destilación probablemente comenzó con la llegada de los españoles y la posterior introducción de alambiques filipinos y árabes en los siglos XVI y XVII (Bruman, 2000; Colunga-GarcíaMarín et al., 2017; Colunga-GarcíaMarín y Zizumbo-Villarreal, 2007; Zizumbo-Villarreal, 1996). No obstante, se ha propuesto que la destilación de agave fermentado ya era practicada por las culturas prehispánicas desde el 400 a.C. (Serra-Puche y Lazcano-Arce, 2016).

La fermentación del mosto cocido de agave para producir destilados emergió de la ancestral conexión entre las plantas de agave, los humanos y los microorganismos. Los productores han

adquirido conocimientos empíricos para dominar el proceso de fermentación, lo cual promueve la proliferación de microorganismos adaptados. Las especies de agave han sido sometidas a una selección constante para aumentar su tamaño y contenido de azúcares, y para reducir la producción de compuestos tóxicos como las saponinas y los cristales de oxalato (Álvarez-Ríos, Pacheco-Torres, et al., 2020). Por ejemplo, *A. tequilana*, frecuentemente utilizada en la producción industrializada de destilados de agave, muestra una reducción del 5 al 32% en el contenido de saponinas en comparación con otras especies de agave (Alcazar-Valle et al., 2019). Las variantes domesticadas de *A. salmiana* también exhiben un tamaño mayor que las variantes salvajes o menos manejadas (Mora-López et al., 2011). Sin embargo, un factor crítico en la domesticación del agave probablemente fue la presión selectiva para obtener propiedades del mosto que facilitaran la fermentación por comunidades microbianas (Colunga-GarcíaMarín et al., 2017). Estas comunidades pueden dispersarse nuevamente en el entorno circundante mediante diversos vectores, facilitando el intercambio de levaduras y bacterias entre ambientes naturales y antropogénicos. Este proceso puede impactar la diversidad microbiana en las fermentaciones de agave (Figura 1).

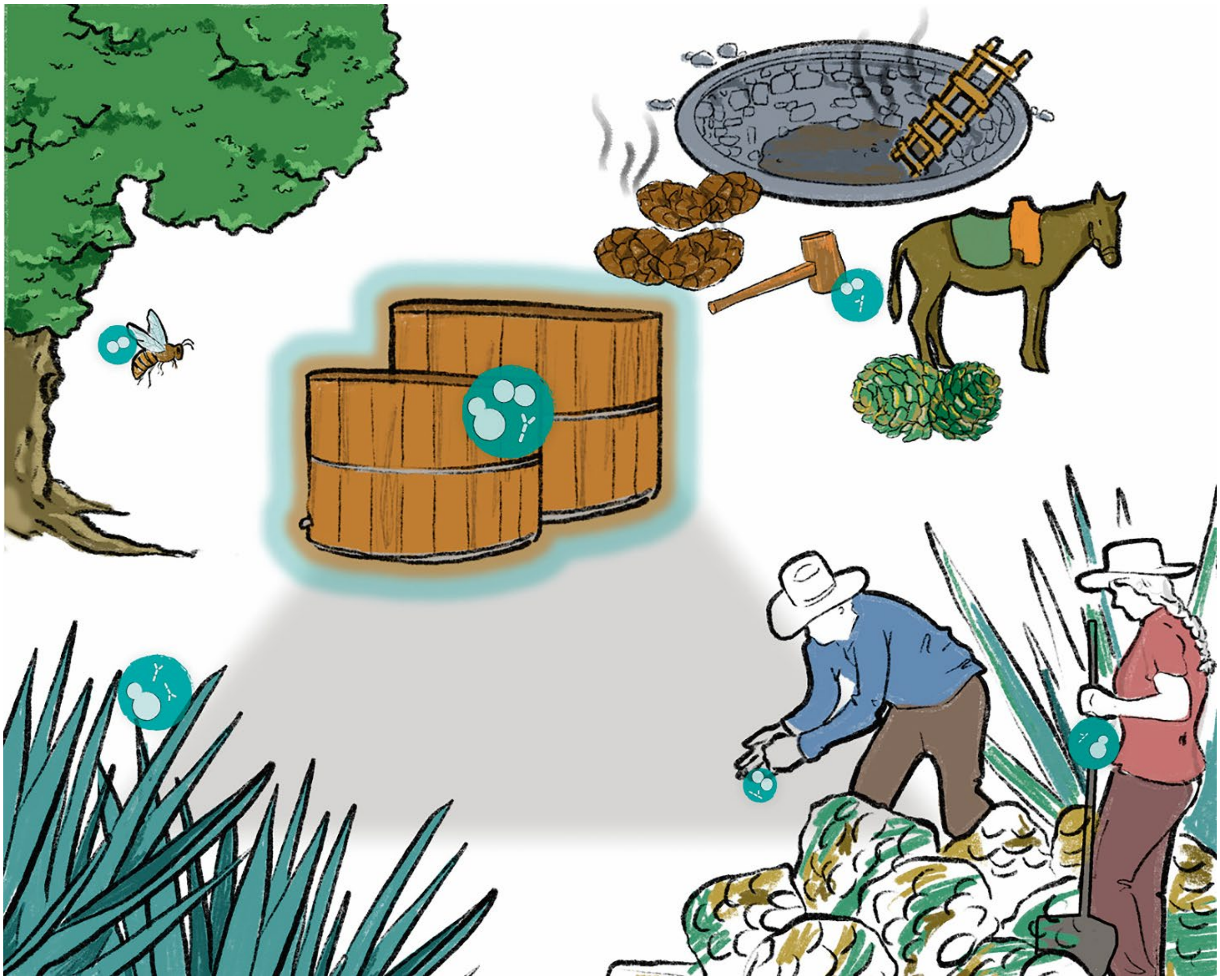

FIGURA 1. Representación de los elementos interconectados que influyen en la comunidad microbiana de la fermentación de agave. Los tres actores clave están conectados por un triángulo: los agaves (izquierda), los productores que dominan el proceso (derecha) y las comunidades microbianas de fermentación abierta (arriba). Los seres humanos han seleccionado de manera selectiva agaves con alto contenido de azúcar y bajos niveles de saponinas. Al mismo tiempo, han elegido comunidades microbianas que son resilientes a compuestos tóxicos, capaces de tolerar la presión osmótica y eficientes en la producción de altos rendimientos de etanol. También se muestran los insectos y otros vectores que facilitan el intercambio de levaduras y bacterias silvestres y asociadas con los humanos entre los entornos naturales y artificiales.

La estandarización del proceso de producción de destilados de agave, impulsada por la expansión del mercado, como en el caso de la industria del tequila, incorporó el uso de cultivos axénicos como inóculos para la fermentación, lo que puede ocasionar una disminución en la diversidad de las comunidades microbianas. La presente revisión se enfoca en las fermentaciones abiertas tradicionales de mosto de agave cocido como un hábitat ecológico para las levaduras que interactúan con una variedad de otros microorganismos.

México es el principal productor de destilados de agave. Desde tiempos antiguos, se han empleado diversas prácticas culturales, combinadas con los recursos naturales disponibles localmente, para elaborar diferentes tipos de destilados de agave (Zizumbo-Villarreal et al., 2009). La cosecha de agaves, su cocción, molienda, fermentación y destilación constituyen los cinco pasos generales principales utilizados en las siete regiones productoras de destilados de agave de México. Los agaves se cosechan tan pronto como están listos para reproducirse sexualmente, ya que en esta etapa se acumulan carbohidratos en el corazón de la planta que se utilizarán para desarrollar el tallo floral. La etapa no reproductiva varía entre especies, desde 5 hasta 15 años (Arellano-Plaza et al., 2022). Una vez que las plantas de agave alcanzan la madurez, se recortan las hojas y se cocina centro o corazón (comúnmente conocido como piña) en hornos de mampostería o en hoyos en la tierra. La cocción facilita la molienda al ablandar las piñas y catalizar la hidrólisis de los fructanos en azúcares fermentables (Mancilla-Margalli y López, 2006; Waleckx et al., 2008). La molienda extrae el jugo y libera los azúcares atrapados en las fibras. Este proceso se realiza utilizando mazos de madera, molinos de piedra (también conocidos como tahonas) o prensas. El mosto de fermentación se obtiene del jugo del agave cocido y triturado, al que se le añade agua. Luego, el mosto se transfiere a diferentes tipos de recipientes, principalmente tanques de madera, mampostería, plástico o acero inoxidable. Ocasionalmente, se emplean otros recipientes como huecos de piedra o sacos de cuero. Posteriormente, comienza la fase de fermentación, que será discutida en las siguientes secciones. Finalmente, el mosto de agave fermentado se destila una o, más comúnmente, dos veces, y el contenido alcohólico se ajusta con agua.

Dado que la mayoría de las producciones a pequeña escala carecen del equipo necesario para controlar las variables fisicoquímicas del proceso, la producción de un destilado artesanal de alta calidad depende del conocimiento empírico y la experiencia de los productores. Esta pericia permite manejar diversos factores que influyen en el aroma de los destilados de agave, como la especie de agave, la edad de la planta, las condiciones de cocción y los procesos de

fermentación (Cedeño-Cruz, 2003; León-Rodríguez et al., 2008; Pinal et al., 2009; Vera-Guzmán et al., 2018; Vera-Guzmán et al., 2010; Vera-Guzmán et al., 2012). El perfil organoléptico de los destilados de agave se atribuye a alcoholes (40-80%), ésteres (8-40%), ácidos (3-26%) y acetales (1-35%), siendo los furfuranos, terpenoides, cetonas, fenoles y aldehídos los responsables del 3-7% restante (Molina-Guerrero et al., 2007). Los furfuranos, piranos, compuestos sulfurosos y cetonas provienen de reacciones de Maillard durante la cocción y la fermentación, mientras que compuestos como el furfural, HMF y la vainillina resultan de la degradación térmica de otros compuestos vegetales (Mancilla-Margalli y López, 2002). Tras la destilación, algunos productores implementan un paso de añejamiento o maduración, lo cual puede alterar significativamente la concentración de compuestos volátiles en los destilados de agave (Acosta-García et al., 2023; Cedeño-Cruz, 2003; López-Ramírez et al., 2013; Mancilla-Margalli y López, 2002).

Los destilados de agave se producen en una variedad de ecosistemas que van desde bosques de roble y caducifolios hasta matorrales xerofíticos (Figura 2A). El área productora se extiende desde la frontera norte de México con los EE.UU. hasta los estados del sur de México, entre los 30°N y 16°S. Este amplio rango abarca áreas con precipitaciones anuales promedio que oscilan entre los 400 mm y los 1,500 mm, temperaturas anuales promedio entre 15 y 25 °C, y altitudes de 110 a más de 2,000 metros sobre el nivel del mar (Figura 2B).

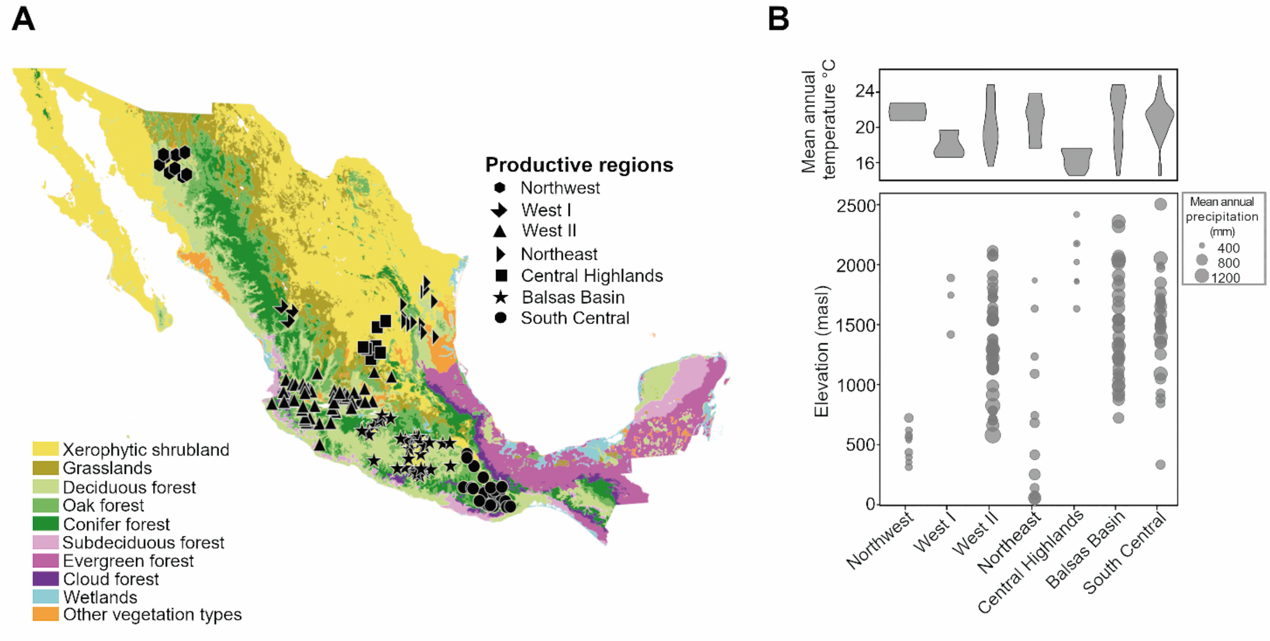

FIGURA 2. Características geográficas y climáticas de las fábricas tradicionales de espirituosos de agave. A) Distribución geográfica de los municipios representativos de la producción tradicional de espirituosos de agave en México. El mapa está codificado por colores según los tipos de vegetación (INEGI, 2003). Los símbolos negros indican la ubicación de estos municipios, y cada figura corresponde a una región productora distinta (Aguirre et al., 2006; Gallegos-Casillas et al., 2024). B) Variables climáticas y ambientales de los municipios, agrupadas por región. Panel superior: Los diagramas de violín muestran la temperatura media anual de los municipios dentro de cada región. Panel inferior: Distribución de altitudes (metros sobre el nivel del mar, msnm) en los municipios dentro de cada región productora. El diámetro de cada círculo representa la precipitación anual media.

Existen siete regiones donde se producen destilados de agave en México: Noroeste, Noreste, Oeste I, Oeste II, Cuenca del Balsas, Altos de Jalisco y Centro-Sur (Aguirre et al., 2006; Gallegos-Casillas et al., 2024). La combinación de características geoclimáticas como la temperatura, las precipitaciones y la altitud varía en cada región productora, por lo que tiene el potencial de contribuir a la singularidad del entorno de fermentación. Además, México es el centro de diversidad del género *Agave*. De las 200 especies de agave que existen en el mundo, 160 se encuentran en México (CONABIO, 2009; García Mendoza, 2002). Actualmente, los destilados de agave se producen a partir de más de 50 taxones de *Agave*, algunos más domesticados que otros (Álvarez-Ainza et al., 2017; Colunga-GarcíaMarín et al., 2017;

Gschaedler et al., 2017; Mora-López et al., 2011). Las especies seleccionadas de agave, junto con otros factores, determinan los cuatro tipos principales de destilados de agave con normas de Denominación de Origen (DO): bacanora, tequila, raicilla y mezcal. Cada tipo se define por las siguientes normas específicas:

i) El bacanora se obtiene específicamente de *Agave angustifolia* y solo se puede producir en el estado noroccidental de Sonora (Gutiérrez-Coronado et al., 2007; Ramírez-Guzmán et al., 2019).

ii) El tequila depende de la variedad azul de *A. tequilana*. El tequila se produce en regiones ubicadas en los estados de Jalisco, Nayarit, Guanajuato y Michoacán, en la región occidental de México, y Tamaulipas, en la frontera noreste del país (Ramírez-Guzmán et al., 2019).

iii) La raicilla se elabora con *Agave maximiliana*, *A. inaequidens*, *A. valenciana*, *A. rhodacantha* y *A. angustifolia*, entre otras especies de agave. La raicilla se produce en una pequeña región situada en los estados occidentales de Jalisco y Nayarit.

iv) El mezcal, cuyo nombre proviene de las palabras náhuatl *metl* que significa “maguey” o agave, e *ixcalli* que significa “asar”, por lo que mezcal significa “agave asado”. A diferencia del bacanora y el tequila, se utilizan varias especies de agave, entre ellas *A. durangensis, A. americana, A. salmiana, A. maximiliana, A. rhodacantha, A. angustifolia, A. cupreata, A. potatorum, A. marmorata, A. karwinskii* y *A. convallis.* La protección por DO abarca los estados de Durango, Zacatecas, San Luis Potosí, Guerrero y Oaxaca, así como algunos municipios en los estados de Tamaulipas, Guanajuato, Puebla, Morelos, Estado de México, Michoacán, Sinaloa y Aguascalientes (Cabrera-Toledo et al., 2020; Colunga-GarcíaMarín y Zizumbo-Villarreal, 2007; CONABIO, 2006, 2009; Espinosa Paz et al., 2005; Jacques-Hernandez et al., 2007; Tello-Balderas y García-Moya, 2017; Vargas-Ponce et al., 2009; Vázquez-Pérez, 2015).

Mientras que las normas de Denominación de Origen (DO) establecen pautas de producción y restringen la elaboración de destilados de agave a áreas geográficas y variedades de agave específicas, en algunas regiones aún persiste una producción artesanal de destilados de agave. Estos se conocen comúnmente como destilados de agave, mezcal o simplemente 'vino' y no siguen ningún estándar oficial. En última instancia, la singularidad de cada entorno de fermentación de agave surge de una combinación única de especies de agave, clima, geografía, comunidades microbianas y prácticas de producción, todos contribuyendo al *terroir* del destilado.

2.1. Las propiedades químicas del mosto de agave, el sustrato de la fermentación

La composición química del sustrato de fermentación del agave, conocido como mosto, está influenciada por las especies de agave y sus técnicas de procesamiento. El mosto es un líquido ácido de color marrón, compuesto por jugo de agave hidrolizado y agua, con cantidades variables de bagazo. El pH reportado del mosto de agave hidrolizado oscila entre 4.0 y 4.8 (Sanchez-Marroquin y Hope, 1953; Waleckx et al., 2008). El mosto es una fuente rica en carbono, con concentraciones de azúcar que van de 14 a 30 °Brix. La fructosa, que constituye hasta el 80% del contenido de azúcares reductores en el mosto de *A. tequilana*, resulta de la hidrólisis térmica de los fructanos del agave, principalmente agavinas (Mancilla-Margalli y López, 2006; Sanchez-Marroquin y Hope, 1953; Waleckx et al., 2008). El contenido de azúcar del mosto presenta fluctuaciones estacionales; por ejemplo, en el mosto de *A. angustifolia*, la concentración de azúcares disminuye de la primavera (295 g/L) al otoño (170 g/L) (Vera-Guzmán et al., 2012). Esta disminución se debe probablemente a la dilución de los azúcares por la mayor absorción de agua durante la temporada de lluvias.

A pesar de ser una fuente rica en carbono, el mosto cocido de agave es limitado en nitrógeno, con una concentración total de aminoácidos cercana a 2.4 mg por litro, lo que representa 130 veces menos que en el aguamiel (la savia del agave) y alrededor de 1,000 veces menos que en el mosto de uva (Díaz-Montaño et al., 2008; Gutiérrez-Gamboa et al., 2017; Ortiz-Basurto et al., 2008; Sanchez-Marroquin y Hope, 1953; Valle-Rodríguez et al., 2012; Waleckx et al., 2008). Las relaciones carbono:nitrógeno reportadas fluctúan entre 69 y 277 (Alcazar-Valle et al., 2019), dependiendo de la especie de agave (Hernández-Cortés et al., 2016; Ortiz-Basurto et al., 2008; Valle-Rodríguez et al., 2012; Vera-Guzmán et al., 2012).

Al igual que el carbono y el nitrógeno, la concentración de compuestos inhibitorios en el sustrato de fermentación, como saponinas, vainillina y furano, depende de la especie de agave y las prácticas de producción. Las saponinas son constituyentes vegetales involucrados en los sistemas de defensa contra insectos y microorganismos. Se ha reportado que estos compuestos tienen efectos inhibitorios sobre el crecimiento de levaduras y bacterias, lo que afecta el rendimiento de la fermentación. Las concentraciones de saponinas en el agave cocido varían de 293 a 431 ppm (Alcazar-Valle et al., 2019). Compuestos como el 5-hidroximetilfurfural (HMF), el

furfural y la vainillina se producen durante el proceso de cocción mediante la degradación de azúcares y lignina (Cedeño-Cruz, 2003; Iwaki et al., 2013; Mancilla-Margalli y López, 2002; Molina-Guerrero et al., 2007). Al final del proceso de cocción, sus concentraciones pueden alcanzar hasta 4,000 ppm para el HMF, 15 ppm para el furfural y 24 ppm para la vainillina (Mancilla-Margalli y López, 2002). Además, otros compuestos presentes en el mosto de agave, como terpenos, aldehídos, furanonas, cetonas, piranos, ácidos orgánicos y compuestos sulfurosos, son conocidos por afectar negativamente el crecimiento microbiano.

Para que la fermentación comience, los productores suelen diluir el jugo cocido de agave con agua, lo que no solo reduce la concentración de azúcares y la presencia de compuestos inhibitorios, sino que también disminuye la presión osmótica. En casos raros, se utilizan aditivos como urea, sulfato de amonio, fosfato de amonio o sulfato de magnesio para complementar las deficiencias de nitrógeno y prevenir una fermentación lenta (Cedeño-Cruz, 2003).

Existe una amplia variedad de prácticas relacionadas con la etapa de fermentación en la producción de destilados de agave. Tradicionalmente, estas fermentaciones son abiertas y comienzan sin la introducción deliberada de un inóculo. Sin embargo, debido a la creciente demanda de destilados, algunas destilerías inoculan con cultivos iniciadores axénicos. A lo largo de este espectro, también existen enfoques intermedios. Algunos productores aceleran el inicio de la fermentación inoculando una comunidad entera de microorganismos en lugar de una cepa aislada.

La fermentación no inoculada depende de microorganismos que pueden ser arrastrados de lotes anteriores que permanecen en los tanques o introducidos desde fuentes cercanas, como las herramientas de la destilería, las instalaciones o fuentes naturales que incluyen animales, suelo y vegetación. Los árboles de roble (*Quercus spp.*), comúnmente encontrados en los bosques cercanos a los sitios de producción de destilados, son hábitats naturales para las levaduras (Kowallik y Greig, 2016; Sampaio y Gonçalves, 2008; Spurley et al., 2021). En México, estos bosques de roble cubren al menos el 4% del territorio y albergan más del 30% de la diversidad mundial de especies de roble (Valencia-Avalos, 2010). Insectos como las moscas de la fruta (*Drosophila*), escarabajos, abejas y avispas también pueden transportar poblaciones de levaduras entre los tanques de fermentación y su entorno (Lachance, 1995; Madden et al., 2018). Aunque se han descrito pocas asociaciones entre levaduras e insectos en este contexto, la

mayoría involucra *S. cerevisiae* y escarabajos, siendo menos documentadas las asociaciones con *Drosophila* (Meriggi et al., 2020).

En algunas destilerías, una pequeña fracción de una fermentación previa o de pulque se utiliza como inóculo para iniciar la fermentación. El pulque se produce fermentando el jugo crudo de agave, conocido como "aguamiel". Dado que no se emplea cocción en su preparación, el microbioma de la planta de agave contribuye a la fermentación. Aunque el microbioma asociado con la fermentación del pulque y las plantas de agave queda fuera del alcance de esta revisión, cabe señalar que este sistema ha sido ampliamente estudiado mediante métodos microbiológicos clásicos y enfoques metagenómicos (Álvarez-Ríos, Figueredo-Urbina, et al., 2020; Astudillo-Melgar et al., 2023; Chacón-Vargas et al., 2020; Enríquez-Salazar et al., 2017; Escalante et al., 2008, 2016, 2004; Lappe-Oliveras et al., 2008; Ojeda-Linares et al., 2021; Rocha-Arriaga y Cruz-Ramirez, 2022; Rocha-Arriaga et al., 2020).

En el otro extremo del espectro, en entornos más industriales, la necesidad de lotes más grandes y uniformes ha llevado a los productores a inocular las fermentaciones de agave con cultivos de levaduras axénicas. El mercado de cepas de levadura adaptadas para destilados de agave ofrece menos opciones en comparación con los de vino o cerveza. Sin embargo, existen cepas específicas para la producción de tequila, y muchos grandes productores de destilados han desarrollado sus propias cepas. Curiosamente, se ha sugerido que los cultivos mixtos de levaduras, en lugar de cepas puras, mejoran la tasa de fermentación, el rendimiento de etanol y los perfiles aromáticos en la producción de destilados de agave, aunque se necesita más investigación en esta área (Acosta-García et al., 2023; González-Robles et al., 2015; Larralde-Corona et al., 2021; Navarrete-Bolaños y Serrato-Joya, 2023; Nuñez-Guerrero et al., 2019). Según lo reportado por diversos productores, el uso de inóculos comerciales sigue siendo poco común en las destilerías tradicionales.

2.2. El microbioma de las fermentaciones de agave

Las bacterias y los hongos microscópicos son algunos de los microorganismos más prevalentes en las fermentaciones de agave. Las bacterias ácido-lácticas (LAB), que incluyen *Lactobacillus brevis, L. casei, L. farraginis, L. kefir, L. plantarum* y *L. pontis*, *Weissella cibaria* y *W. paramesenteroides*, constituyen los principales grupos bacterianos identificados en estas fermentaciones (Escalante-Minakata et al., 2008; Kirchmayr et al., 2017). La fermentación de

agave por LAB conduce a la acumulación de ácido láctico, lo que acidifica el ambiente (Narváez-Zapata et al., 2010) y puede influir en la proliferación de levaduras (Escalante et al., 2016; Lappe-Oliveras et al., 2008). Las comunidades compuestas por levaduras y LAB son comunes en bebidas alcohólicas tradicionales fermentadas a partir de maíz, coco, piña o tuna en México (Ojeda-Linares et al., 2021), así como en bebidas alcohólicas hechas de arroz fermentado, malta o manzanas, como el sake, la cerveza y la sidra en otras partes del mundo (Bokulich et al., 2014; Tyakht et al., 2021). Se ha sugerido que la presencia simultánea de LAB y levaduras podría estimular cambios metabólicos en ambos microorganismos, generando compuestos que influyen en las características organolépticas del producto final (De Vuyst y Leroy, 2020; Narváez-Zapata et al., 2010; Narvhus y Gadaga, 2003).

Las bacterias ácido-acéticas (AAB) de los géneros *Acetobacter* y *Gluconobacter* son otro grupo prominente identificado en las fermentaciones de agave (Escalante-Minakata et al., 2008; Kirchmayr et al., 2017). La presencia de AAB puede influir en el proceso general de fermentación al afectar el equilibrio de las poblaciones microbianas y contribuir a la producción de ácidos orgánicos, que sirven como sustratos para la formación de ciertos compuestos volátiles. *Zymomonas mobilis*, conocida por su capacidad para producir etanol, también es prevalente en las fermentaciones de agave (Escalante-Minakata et al., 2008; Kirchmayr et al., 2017). También se han detectado bacterias formadoras de esporas, cuya presencia se puede atribuir al contacto entre las piñas cocidas y el suelo o el equipo utilizado para el molino (Kirchmayr et al., 2017).

Numerosos estudios se han centrado en identificar la composición de levaduras del microbioma del agave cocido. Una de las primeras contribuciones importantes fue realizada hace más de 25 años por Marc-André Lachance, quien llevó a cabo un estudio exhaustivo sobre la fermentación de agave en una destilería tradicional de tequila utilizando métodos clásicos para la clasificación de levaduras. Lachance tomó muestras de todas las etapas del proceso de producción del tequila, desde la cosecha de la planta de agave hasta la fermentación, e incluso recogió especímenes de *Drosophila* en las cercanías del sitio de producción (Lachance, 1995). Este estudio concluyó que las especies endógenas de levaduras encontradas en la planta de agave, como *Clavispora lusitaniae* y *Metschnikowia agaves*, difieren de las presentes en los tanques de fermentación, donde *S. cerevisiae, Maudiozyma humilis* y *Brettanomyces anomalus* fueron identificadas como las especies predominantes.

Más de 50 especies de levaduras han sido aisladas de las fermentaciones tradicionales de agave provenientes de 15 especies diferentes de sustratos de agave. La Figura 3 proporciona una visión general de las especies de levaduras reportadas en este entorno (Aldrete-Tapia et al., 2018; Aldrete-Tapia et al., 2020; Álvarez-Ainza et al., 2015; Arias-García, 2008; Díaz-Montaño et al., 2008; Escalante-Minakata et al., 2008; Espinoza-Martinez et al., 2023; Gallegos-Casillas et al., 2024; García-Ortega et al., 2022; Garibay-Marcelo, 2019; Gómez-Márquez et al., 2022; Kirchmayr et al., 2017; Lachance, 1995; Martínez-Estrada et al., 2024; Nolasco-Cancino et al., 2018; Páez-Lerma et al., 2013; Peris et al., 2023; Peter et al., 2018; Verdugo Valdez et al., 2011). En la mayoría de las fermentaciones de agave, se observa que levaduras no *Saccharomyces,* como *T. delbrueckii, K. marxianus, P. kluyveri* y *Hanseniaspora spp.,* proliferan durante las primeras etapas. A medida que avanza la fermentación y aumentan los niveles de etanol, estas levaduras son superadas por especies con mayor tolerancia al etanol, como *S. cerevisiae* (Garibay-Marcelo, 2019; Kirchmayr et al., 2017; Lachance, 1995; Nolasco-Cancino et al., 2018; Páez-Lerma et al., 2013; Verdugo Valdez et al., 2011; Walker et al., 2019).

Estudios exhaustivos sobre la diversidad microbiana en las fermentaciones tradicionales de agave en destilerías de las siete regiones productoras de espíritus de agave en México revelan que las comunidades fúngicas permanecen relativamente consistentes a lo largo del proceso de fermentación (Gallegos-Casillas et al., 2024; Jara-Servin et al., 2025). Estos estudios también identificaron un grupo central de especies fúngicas que son prominentes en estas fermentaciones, siendo *S. cerevisiae, T. delbrueckii, K. marxianus* y varias especies de los géneros *Pichia, Zygosaccharomyces* y *Hanseniaspora* las más abundantes. Entre estas especies centrales, tres especies de hongos filamentosos, *Penicillium polonicum, Mycosphaerella tassiana* y *Aureobasidium pullulans*, fueron identificadas por primera vez, ya que no se habían reportado previamente en este entorno de fermentación (Jara-Servin et al., 2025). Las levaduras que se aislaron con menor frecuencia incluyen especies de los géneros *Candida, Kazachstania* y *Rhodotorula* (Gallegos-Casillas et al., 2024) (Figura 3). Jara-Servin y sus colegas también identificaron más de 200 especies fúngicas que no se habían asociado previamente con ambientes de fermentación de agave. De estas, el 81% pertenecen al filo Ascomycota, el 17% a Basidiomycota, y el 2% restante consiste en especies de los filos Mortierellomycota y Mucoromycota. Este hallazgo amplía nuestra comprensión de la diversidad fúngica presente en la fermentación de agave, destacando los complejos ecosistemas microbianos que contribuyen a la producción de los espíritus de agave (Jara-Servin et al., 2025).

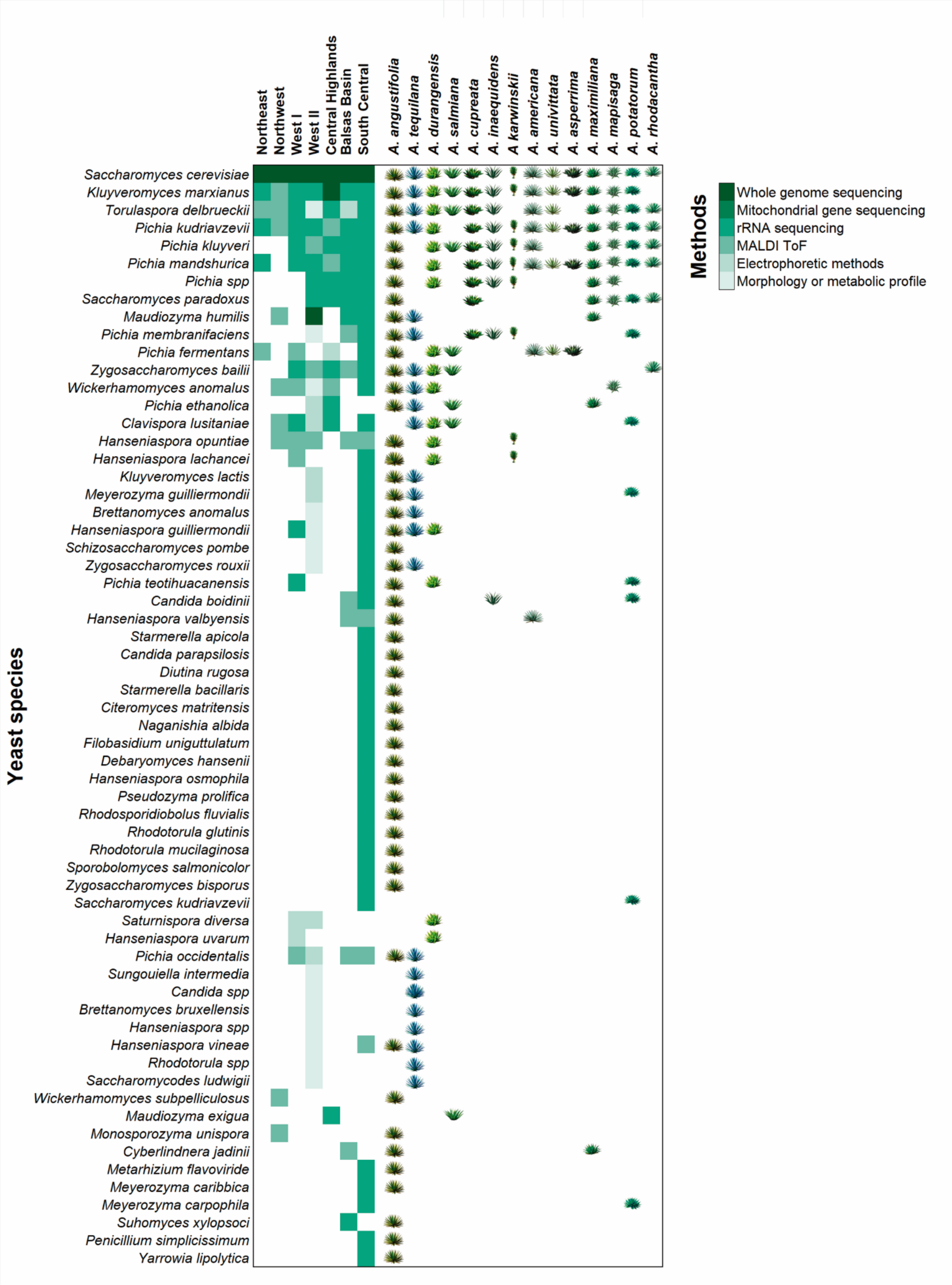
Northeast
Northwest
West I
West II
Central Highlands
Balsas Basin
South Central
A. angustifolia
A. tequilana
A. durangensis
A. salmiana
A. cupreata
A. inaequidens
A karwinskii
A. americana
A. univittata
A. asperrima
A. maximiliana
A. mapisaga
A. potatorum
A. rhodacantha
Methods
Whole genome sequencing
Mitochondrial gene sequencing
rRNA sequencing
MALDI ToF
Electrophoretic methods
Morphology or metabolic profile
Yeast species
Saccharomyces cerevisiae
Kluyveromyces marxianus
Torulaspora delbrueckii
Pichia kudriavzevii
Pichia kluyveri
Pichia mandshurica
Pichia spp
Saccharomyces paradoxus
Maudiozyma humilis
Pichia membranifaciens
Pichia fermentans
Zygosaccharomyces bailii
Wickerhamomyces anomalus
Pichia ethanolica
Clavispora lusitaniae
Hanseniaspora opuntiae
Hanseniaspora lachancei
Kluyveromyces lactis
Meyerozyma guilliermondii
Brettanomyces anomalus
Hanseniaspora guilliermondii
Schizosaccharomyces pombe
Zygosaccharomyces rouxii
Pichia teotihuacanensis
Candida boidinii
Hanseniaspora valbyensis
Starmerella apicola
Candida parapsilosis
Diutina rugosa
Starmerella bacillaris
Citeromyces matritensis
Naganishia albida
Filobasidium uniguttulatum
Debaryomyces hansenii
Hanseniaspora osmophila
Pseudozyma prolifica
Rhodosporidiobolus fluvialis
Rhodotorula glutinis
Rhodotorula mucilaginosa
Sporobolomyces salmonicolor
Zygosaccharomyces bisporus
Saccharomyces kudriavzevii
Saturnispora diversa
Hanseniaspora uvarum
Pichia occidentalis
Sungouiella intermedia
Candida spp
Brettanomyces bruxellensis
Hanseniaspora spp
Hanseniaspora vineae
Rhodotorula spp
Saccharomycodes ludwigii
Wickerhamomyces subpelliculosus
Maudiozyma exigua
Monosporozyma unispora
Cyberlindnera jadinii
Metarhizium flavoviride
Meyerozyma caribbica
Meyerozyma carpophila
Suhomyces xylopsoci
Penicillium simplicissimum
Yarrowia lipolytica

FIGURA 3. Diversidad de levaduras en las fermentaciones tradicionales de agave en México. Para proporcionar una comprensión completa de la diversidad de levaduras en este entorno, estructuramos los datos basados en las siete regiones productoras de espirituosos de agave reportadas en (Gallegos-Casillas et al., 2024). Izquierda: El mapa de calor (escala de color verde) indica el método de identificación de levaduras utilizado; solo se muestra el método con mayor precisión para cada especie por región. Estos métodos incluyen perfiles morfológicos y metabólicos, PCR-RFLP, MALDI-ToF, secuenciación de genes rRNA, secuenciación de genes mitocondriales y secuenciación de genoma completo. Derecha: Presencia de especies de levaduras en el mosto de agave cocido extraído de las especies de agave representadas o mezclas que contienen dichas especies. Las ilustraciones de agave son de Rafael Ruíz (CONABIO, 2006).

Dada su presencia generalizada en las fermentaciones de agave, nos centraremos en describir las características reportadas en aislados de *S. cerevisiae*, *K. marxianus*, *T. delbrueckii* y *Pichia spp*. de este ambiente.

2.2.1. Saccharomyces cerevisiae

Saccharomyces cerevisiae ha sido aislada de cada una de las etapas de la fermentación de mosto de todas las especies de agave que han sido estudiadas (Figura 3) (Aldrete-Tapia et al., 2018; Aldrete-Tapia et al., 2020; Álvarez-Ainza et al., 2015; Arias-García, 2008; Avelar-Rivas et al., 2024; Díaz-Montaño et al., 2008; Escalante-Minakata et al., 2008; Espinoza-Martínez et al., 2023; Gallegos-Casillas et al., 2024; Garibay-Marcelo, 2019; Jara-Servin et al., 2025; Kirchmayr et al., 2017; Lachance, 1995; Martínez-Estrada et al., 2024; Nolasco-Cancino et al., 2018; Páez-Lerma et al., 2013; Peris et al., 2023; Peter et al., 2018; Verdugo Valdez et al., 2011). Se ha reportado que las cepas de *S. cerevisiae* exhiben una alta diversidad genética entre las destilerías dentro de un mismo estado, e incluso entre los tanques de fermentación de una misma destilería (Aldrete-Tapia et al., 2018; Álvarez-Ainza et al., 2015; Páez-Lerma et al., 2013). Algunos estudios genómicos han analizado más de 200 cepas de *S. cerevisiae* provenientes de las siete regiones reconocidas de producción de bebidas destiladas de agave en México. Los resultados de estos estudios indican que la mayoría de las cepas de *S. cerevisiae* provenientes de fermentaciones de agave se agrupan en una línea genética distinta, que comparte un ancestro común con otras cepas neotropicales, como las cepas asociadas al tracto intestinal humano de la Guayana Francesa y cepas silvestres de Brasil y Ecuador (Avelar-Rivas et al.,

2024; Peter et al., 2018; Pontes et al., 2020). Avelar-Rivas y colaboradores también reportaron que una minoría de los aislamientos provenientes de fermentaciones de agave (n=5) se agruparon con el clado de roble norteamericano, junto con una cepa migrante que se asoció al clado del vino.

Antes de la existencia de este valioso recurso de más de 200 genomas de *S. cerevisiae* provenientes de fermentaciones de agave, las evidencias provenientes de marcadores mitocondriales sugerían fuertemente la existencia de diversas poblaciones a lo largo del país (Páez-Lerma et al., 2013). El análisis de todo el genoma (Avelar-Rivas et al., 2024) confirmó la alta diversidad genética en los aislados de *S. cerevisiae* provenientes de agave en México, identificando una estructura principal de estas cepas en diez poblaciones dispuestas en tres clados filogenéticos diferentes: Agave Mexicano 1, Agave Mexicano 2 y Destilería de Tequila. Además, la diferenciación entre Agave Mexicano 1 y 2 se correlaciona con su aislamiento debido a la barrera geográfica representada por la cadena montañosa conocida como Sierra Madre Oriental. Adicionalmente, las cepas provenientes del lado occidental de la Sierra Madre Oriental muestran un gradiente de diversidad genética de norte a sur, siendo las cepas aisladas de latitudes más bajas más diversas (Avelar-Rivas et al., 2024).

Las cepas de Agave Mexicano 1 parecen exhibir signos de domesticación, incluyendo numerosas regiones con pérdida de heterocigosidad, una mayor frecuencia de marcos de lectura abierta (ORFs) con variantes en el número de copias, altos niveles de heterocigosidad a través del genoma y la presencia de la endonucleasa de homing VDE en el gen *VMA1* (O'Donnell et al., 2023; Peter et al., 2018). Esta versión del gen VMA1 está ausente en las poblaciones silvestres de *S. cerevisiae*, pero se encuentra presente en las cepas domesticadas utilizadas tanto en fermentación en estado sólido como líquido (Han et al., 2021). Estas cepas también contienen un alelo del gen *RTM1* asociado con la utilización de sacarosa y la resistencia a compuestos inhibitorios presentes en la melaza. Además, han perdido acuaporinas funcionales en la membrana, lo cual se hipotetiza que es una consecuencia de la domesticación asociada a la transición a nichos de alta concentración de azúcar (Pontes et al., 2020).

Es relevante señalar que las cepas de *S. cerevisiae* provenientes de agave muestran una proporción inusualmente alta de genes de la especie hermana *S. paradoxus*, los cuales provienen de varios pulsos de introgressión de linajes americanos (Avelar-Rivas et al., 2024; Peter et al., 2018; Tellini et al., 2024). Estos genes incluyen los alelos de *S. paradoxus* de

BIO1/BIO6, que codifican enzimas involucradas en la síntesis de biotina (Pontes et al., 2020). Es importante destacar que *S. paradoxus* también ha sido aislada en el mosto de fermentación de siete especies diferentes de agave en cinco regiones productoras en México (Figura 3) (Gallegos-Casillas et al., 2024). Esto sugiere que las interacciones inter-especies de *Saccharomyces* son relevantes dentro del entorno de fermentación de agave.

Datos experimentales también han revelado posibles rasgos adaptativos en las cepas de *S. cerevisiae* provenientes del entorno de fermentación de agave. Las cepas autóctonas de *S. cerevisiae*, aisladas de las destilerías de bebidas espirituosas de agave, pueden crecer a 42°C, lo que les otorga una ventaja en las fermentaciones de agave, donde la temperatura no se controla y puede alcanzar hasta 40°C dentro de los tanques (Ruiz-Terán et al., 2019). La floculación, que podría involucrar cambios morfológicos en las paredes celulares, parece contribuir a esta termotolerancia. De manera destacada, los genes relacionados con la floculación *FLO1, FLO5* y *FLO11* se sobreexpresan en una cepa aislada de una fermentación de agave, en comparación con una cepa comercial de vino (Vergara-Álvarez et al., 2019). Además, los genes *OLE1, OLE2, ERG1, ERG11* y *ERG25*, involucrados en el metabolismo del ácido oleico y el ergosterol, mostraron una expresión diferencial en una cepa de *S. cerevisiae* aislada de una fermentación de tequila, en comparación con una cepa de laboratorio (Ramirez-Córdova et al., 2012). Estos genes podrían estar asociados con la tolerancia al etanol, ya que las concentraciones en la fermentación de agave típicamente varían entre 4 y 9% (Cedeño-Cruz, 2003). Finalmente, una cepa de *S. cerevisiae* de agave mostró un mejor rendimiento en la fermentación de sustratos con alto contenido de azúcar, como el mosto de agave, en comparación con cepas provenientes de mosto de uva (Arrizon et al., 2006). Esto sugiere que las cepas de *S. cerevisiae* provenientes de la fermentación de agave podrían, de hecho, haberse adaptado a este entorno.

2.2.2. Kluyveromyces marxianus

Kluyveromyces marxianus es una levadura aeróbica que, predominantemente, depende de un metabolismo respiro-fermentativo para la producción de energía (Lane y Morrissey, 2010). A diferencia de *Saccharomyces cerevisiae*, algunas cepas de *K. marxianus* tienen la capacidad de utilizar lactosa o inulina como fuentes de carbono. Esta levadura ha sido identificada en la fermentación tradicional de mostos de agave de trece especies de agave utilizadas en la producción de bacanora, mezcal y tequila en diversas regiones de México (Figura 3) (Aldrete-

Tapia et al., 2020; Arias-García, 2008; Escalante-Minakata et al., 2008; Gallegos-Casillas et al., 2024; Garibay-Marcelo, 2019; Gómez-Márquez et al., 2022; Kirchmayr et al., 2017; Lachance, 1995; Martínez-Estrada et al., 2024; Nolasco-Cancino et al., 2018; Páez-Lerma et al., 2013; Verdugo Valdez et al., 2011).

Hasta la fecha, se han reportado tres secuencias genómicas de *K. marxianus* aisladas de fermentaciones de agave. La primera proviene de Sudáfrica (Schabort et al., 2016), mientras que las otras dos son de México: una de la región de los Altos de Jalisco y otra del mosto fermentado de *Agave fourcroydes* en la península de Yucatán (Gómez-Márquez et al., 2022; Lappe-Oliveras et al., 2023; Lozano-Aguirre et al., 2024). Dado que las regiones de Yucatán y Sudáfrica no son históricamente conocidas por la producción de destilados de agave, estas cepas no están incluidas en el inventario presentado en la Figura 3.

El análisis filogenético muestra que la cepa sudafricana forma una línea única y altamente divergente, distinta tanto de las cepas de fermentación láctea (haplotipos B) como de las no lácteas (haplotipos A) (Ortiz-Merino et al., 2018). Generalmente, las cepas de fermentación láctea son diploides o triploides, mientras que las de fermentación no láctea son haploides, lo que sugiere una relación entre la fuente de aislamiento y la ploidía. Curiosamente, la cepa mexicana de los Altos de Jalisco es diploide y contiene tanto haplotipos A como B (Gómez-Márquez et al., 2022). Para comprender la relación filogenética entre las cepas de agave y lácteas, se requieren más análisis genómicos de cepas adicionales provenientes de fermentaciones de agave.

Varias cepas de *K. marxianus* aisladas de mostos de agave muestran características indicativas de adaptación a este entorno de fermentación, como una mayor tolerancia al etanol, resistencia a los saponinas y una mayor capacidad de asimilación de fructanos (Alcázar-Valle, 2016). A pesar de algunos reportes sobre alta sensibilidad al etanol, *K. marxianus* suele ser la especie no-*Saccharomyces* predominante en las etapas finales de la fermentación del agave (Álvarez-Ainza et al., 2021; Lachance, 1995; Verdugo Valdez et al., 2011). Su mayor tolerancia a las saponinas, en comparación con *S. cerevisiae*, se debe a una mayor concentración de 1,3 ß-glucanos y mananos en su pared celular, así como a la inducción de enzimas hidrolizantes de saponinas (Alcázar-Valle, 2016; Alcázar-Valle et al., 2019). Además, las cepas de *K. marxianus* de fermentaciones de agave muestran una mayor actividad de fructanasa en comparación con otras

levaduras, lo que podría mejorar su capacidad para asimilar fructanos de los hidrolizados de agave (Arrizon et al., 2012).

Debido a la alta variabilidad fenotípica entre las cepas de *K. marxianus*, determinar si esta especie tiene una capacidad fermentativa superior en comparación con *S. cerevisiae* sigue siendo un desafío. Algunos estudios sugieren que las cepas de *K. marxianus* producen más etanol en la fermentación de mosto de agave (96% frente a 70%), aunque estos hallazgos parecen ser dependientes de la cepa (Adame-Soto et al., 2023; Amaya-Delgado et al., 2013; López-Álvarez et al., 2012). Independientemente de la cepa, *K. marxianus* tiende a producir más compuestos volátiles, particularmente ésteres y alcoholes superiores como isoprenol, 3-metilpentanol, linalool, nerolidol y timol, en comparación con *S. cerevisiae* (Amaya-Delgado et al., 2013; López-Álvarez et al., 2012; Segura-García et al., 2015). Las fermentaciones de agave con cultivos mixtos de levaduras, incluyendo *K. marxianus*, presentan un perfil más amplio de compuestos aromáticos (Navarrete-Bolaños y Serrato-Joya, 2023).

2.2.3. Torulaspora delbrueckii

Torulaspora delbrueckii ha sido aislada de una amplia gama de sustratos, incluyendo frutas, insectos, suelos, plantas, agua de mar, alimentos en descomposición y mostos de agave (Kurtzman, 2011). Esta especie ha sido documentada en fermentaciones de doce especies de agave a lo largo de todas las regiones productoras de agave en México (Espinoza-Martínez et al., 2023; Gallegos-Casillas et al., 2024; Garibay-Marcelo, 2019; Kirchmayr et al., 2017; Lachance, 1995; Martínez-Estrada et al., 2024; Páez-Lerma et al., 2013; Verdugo Valdez et al., 2011). En el mosto de *Agave salmiana*, esta levadura está presente tanto en las primeras como en las últimas etapas de la fermentación (Álvarez-Ainza et al., 2021; Lachance, 1995; Verdugo Valdez et al., 2011), y su presencia en algunas destilerías fluctúa en respuesta a los cambios de temperatura (Martínez-Estrada et al., 2024).

A diferencia de *S. cerevisiae, T. delbrueckii* puede mantener un metabolismo respiratorio en condiciones de bajo oxígeno, lo cual probablemente impacta su capacidad fermentativa (Fernandes et al., 2021). Sin embargo, al igual que otras levaduras, existe una variabilidad significativa en la capacidad fermentativa y la tolerancia al etanol entre las distintas cepas (Fernandes et al., 2021; Kurtzman, 2011; Nuñez-Guerrero et al., 2016).

Como otras levaduras no-*Saccharomyces*, *T. delbrueckii* contribuye a la mejora de los perfiles aromáticos y de sabor en bebidas alcohólicas a través de la producción de compuestos volátiles. Su rol ha sido ampliamente estudiado en cervezas y vinos, donde se asocia con un mayor aumento en la producción de alcoholes superiores (Canonico et al., 2017; Tufariello et al., 2021). Las bebidas destiladas de agave producidas con cultivos mixtos de *S. cerevisiae* y *T. delbrueckii* presentan perfiles enriquecidos de ésteres y terpenos, mejorando las propiedades sensoriales de los destilados (Nuñez-Guerrero et al., 2016).

2.2.4. Pichia spp.

Las especies del género *Pichia* son levaduras comunes en la fermentación de leche agria, carne, queso ácido, aceitunas, cacao y café (Tofalo et al., 2020). Se encuentran en una gran variedad de ambientes naturales, incluyendo frutas, frutas en descomposición, tejidos vegetales, esputo humano y animales. Varias especies de *Pichia* han sido aisladas de fermentaciones de catorce especies de agave a lo largo de todas las regiones productoras de agave, siendo *P. kudriavzevii*, *P. kluyveri* y *P. manshurica* las más comúnmente encontradas (Figura 3) (Aldrete-Tapia et al., 2020; Arias-García, 2008; Escalante-Minakata et al., 2008; Gallegos-Casillas et al., 2024; Garibay-Marcelo, 2019; Kirchmayr et al., 2017; Lachance, 1995; Nolasco-Cancino et al., 2018; Páez-Lerma et al., 2013; Verdugo Valdez et al., 2011). *P. kudriavzevii* y *P. manshurica* son más frecuentes en las primeras etapas de la fermentación de destilados de agave (Nolasco-Cancino et al., 2018), mientras que *P. membranifaciens* se ha encontrado en todas las etapas de la fermentación del tequila (Lachance, 1995). La presencia de *P. kluyveri* a menudo se asocia con fluctuaciones de temperatura durante la fermentación (Martínez-Estrada et al., 2024). Dos posibles nuevas especies de *Pichia* aisladas de fermentaciones de agave fueron reportadas por Gallegos-Casillas et al. (2024); recientemente una de estas especies fue descrita formalmente como *Pichia teotihuacanensis* (Chai et al., 2024).

La investigación sobre los mecanismos que mejoran el rendimiento de etanol y los perfiles de sabor en cultivos mixtos de levaduras que incluyen especies de *Pichia* ha ganado un interés creciente. Por ejemplo, se encontró que el consumo de azúcar de *P. kudriavzevii* fue el doble cuando se cultivó en co-cultivo con *S. cerevisiae* o *K. marxianus* en comparación con su cultivo puro, lo que podría contribuir a una mayor producción de etanol. Además, la presencia de *P. kudriavzevii* se ha asociado con concentraciones más altas de alcoholes superiores, lo que puede afectar positiva o negativamente el aroma y el sabor (Liu et al., 2016; Nolasco-Cancino et

al., 2018). En la fermentación de tequila, *P. kluyveri* produce una mayor cantidad de ésteres en comparación con *S. cerevisiae*, lo que mejora la fragancia de los destilados (Amaya-Delgado et al., 2013; Méndez-Zamora et al., 2021; Segura-García et al., 2015). Estos hallazgos subrayan el papel de las levaduras no-*Saccharomyces* en la mejora del sabor y aroma de los espirituosos de agave.

2.3. Otras especies

Además de las especies descritas anteriormente, las levaduras del género *Hanseniaspora* han llamado la atención por su capacidad para producir una amplia gama de compuestos aromáticos. Varias especies de *Hanseniaspora* han sido identificadas en fermentaciones de mostos de *Agave angustifolia*, *A. durangensis*, *A. tequilana*, *A. karwinskii* y *A. americana* en las regiones productoras Noroeste I, Oeste I, Oeste II, Cuenca del Balsas, Central Sur (Figura 3) (Arias-García, 2008; Díaz-Montaño et al., 2008; Gallegos-Casillas et al., 2024; Kirchmayr et al., 2017; Lachance, 1995; Martínez-Estrada et al., 2024; Páez-Lerma et al., 2013).

Además, se han identificado especies de *Maudiozyma* y *Monosporozyma* en fermentaciones de *A. angustifolia, A. maximiliana, A. tequilana* y *A. salmiana* de diversas regiones (Gallegos-Casillas et al., 2024; García-Ortega et al., 2022; Lachance, 1995; Verdugo Valdez et al., 2011). Los estudios genómicos de *Maudiozyma humilis* (anteriormente clasificada como *Kazachstania humilis*) de agave revelaron que estas cepas forman un clúster distinto, divergente de otras cepas de masa madre. Esta divergencia está respaldada por reordenamientos genómicos y variantes estructurales, que probablemente resultaron de la adaptación a las condiciones específicas de la fermentación de agave. Por ejemplo, en una cepa de *M. humilis* derivada de agave se identificó una duplicación del gen *ZWF1*, que en *S. cerevisiae* está asociado con la tolerancia al furano (Gallegos-Casillas et al., 2024).

En resumen, diversas especies de levaduras se encuentran comúnmente en fermentaciones de agave, y sus análisis genómicos revelan grupos filogenéticos distintos, separándolas de las cepas aisladas de vino, cerveza u otros tipos de fermentación (Avelar-Rivas et al., 2024; 2011; Gallegos-Casillas et al., 2024; Páez-Lerma et al., 2013). Sin embargo, debido a la compleja composición química de los mostos en fermentación, los rasgos adaptativos pueden variar incluso entre cepas de la misma especie, lo que resulta en una notable diversidad metabólica dentro de estas comunidades microbianas especializadas. Se requiere más investigación para

comprender completamente las contribuciones e interacciones de cada cepa y especie en la fermentación de agave.

3. CONCLUSIONES Y PERSPECTIVAS

Los destilados de agave, profundamente arraigados en la cultura mexicana, han ganado una apreciación global debido a la riqueza de sus procesos de elaboración y sus perfiles organolépticos distintivos. Estos destilados, obtenidos principalmente a través de fermentaciones no inoculadas, dependen de un consorcio microbiano de levaduras y bacterias que prosperan en la interfaz entre los ambientes naturales y humanos. La práctica de la fermentación no inoculada del mosto de agave, que data de las culturas prehispánicas, sugiere que las comunidades microbianas en este hábitat han evolucionado características específicas para prosperar en ambientes con alto contenido de azúcar y compuestos inhibitorios. A pesar de esta larga historia, aún no se comprende completamente el alcance de la contribución de la comunidad microbiana y sus actividades enzimáticas específicas al bouquet de los destilados de agave. Las vías metabólicas involucradas en la producción de compuestos organolépticos se han descrito extensamente por Dzialo y colaboradores (Dzialo et al., 2017). Sin embargo, otros factores, como la interacción entre diferentes especies microbianas y la relación entre las propiedades químicas de los sustratos y los microorganismos, también pueden jugar un papel crucial en la determinación del perfil sensorial final de los destilados de agave. Se requieren estudios sistemáticos que comparen los perfiles organolépticos de fermentaciones de agave utilizando las mismas comunidades microbianas cultivadas en diferentes sustratos de agave para entender cómo la composición química del mosto de agave influye en el sabor y el aroma. A su vez, comparar los perfiles organolépticos producidos por diferentes comunidades microbianas fermentando el mismo sustrato de agave ayudaría a esclarecer el complejo papel de las interacciones microbianas y sus contribuciones relativas al aroma y al sabor.

Las fermentaciones de agave ocurren en diversos ecosistemas, y debido a su naturaleza artesanal, cada destilería emplea una combinación única de prácticas en los cinco pasos necesarios para producir los destilados. Estos factores influyen en las comunidades microbianas fúngicas de las fermentaciones de agave, que parecen estar principalmente determinadas por las características locales y las prácticas de cada sitio de producción; la propia destilería juega un papel importante en dar forma a la diversidad de las comunidades bacterianas y fúngicas (Jara-Servín et al., 2025). Sin embargo, estos estudios se han centrado principalmente en la diversidad

a nivel de especie, y aún falta determinar si la diversidad fúngica intraespecífica observada está correlacionada con la distribución geográfica.

Los esfuerzos de muestreo a gran escala y secuenciación dirigidos a las levaduras de las fermentaciones de agave han enriquecido nuestra comprensión de sus orígenes ecológicos, relaciones filogenéticas y dinámicas evolutivas, particularmente para la especie modelo, *S. cerevisiae.* Estos estudios revelan una diversidad significativa y múltiples eventos de introgresión de su especie hermana, *S. paradoxus*. Sin embargo, los estudios similares enfocados en otras especies de levaduras de *Saccharomyces* y no *Saccharomyces,* que también juegan papeles cruciales en la fermentación del agave cocido, siguen siendo escasos.

El creciente aumento de la demanda global representa una amenaza para los métodos tradicionales de producción de espirituosos de agave y, en consecuencia, para la diversidad tanto de las plantas de agave como de los microorganismos asociados. La investigación sobre las características genómicas y fenotípicas de toda la comunidad de levaduras de las fermentaciones de agave profundizará nuestro entendimiento de las dinámicas microbianas y la evolución. Este conocimiento podría aplicarse para mejorar la eficiencia y sostenibilidad de la producción industrial, a la vez que se preservan los recursos naturales involucrados.

DECLARACIÓN DE CONTRIBUCIONES DE LOS AUTORES

M. Colón-González y L. Morales realizaron la búsqueda primaria de literatura y la recopilación de datos. A. DeLuna, E. Mancera, X. Aguirre-Dugua, M. G. Guerrero-Osornio y J. A. Avelar-Rivas asistieron con búsquedas adicionales y el análisis de datos. M. Colón-González y L. Morales redactaron el manuscrito, mientras que todos los autores contribuyeron a la redacción de secciones específicas y a la creación de las figuras. Todos los autores participaron en la revisión del borrador final.

AGRADECIMIENTOS

Agradecemos a Carina Uribe-Díaz (LIIGH-UNAM), Alejandra Castillo (LIIGH-UNAM), Luis Aguilar (LAVIS-UNAM) y Jair García (LIIGH-UNAM) por su apoyo en la tecnología de la información utilizada para completar este manuscrito. A Manuel R. Kirchmayr por sus útiles discusiones sobre las levaduras de la fermentación de agave y a Diego Ortega-Del Vecchyo por sus discusiones

sobre la domesticación. Este trabajo fue apoyado por CONACYT [números de proyecto CB-2016-01 284992, FORDECYT-PRONACES/103000/2020, CF-2023-G-695], por PAPIIT-DGAPA-UNAM [números de proyecto IN209021, IN212524] y por el proyecto SEP-CINVESTAV [número 23]. M. Colón-González recibe actualmente una beca postdoctoral de CONACYT bajo el programa Estancias Postdoctorales por México 2022. M. G. Guerrero-Osornio es estudiante de doctorado en el Posgrado en Ciencias Biológicas de la Universidad Nacional Autónoma de México (UNAM) y recibe la beca 2023-000002-01NACF-03323 de CONACYT.

DECLARACIÓN DE CONFLICTO DE INTERÉS

Los autores declaran no tener conflicto de interés.

DECLARACIÓN DE DISPONIBILIDAD DE DATOS

El intercambio de datos no es aplicable a este artículo, ya que no se crearon ni analizaron nuevos datos en este estudio.

REFERENCIAS

Acosta-García, Erick D., Jesús B. Páez-Lerma, Martha R. Moreno-Jiménez, Juan A. Rojas-Contreras, and Nicolás O. Soto-Cruz. 2023. "Yeast Competition during Alcoholic Fermentation of Agave. Its Comprehension as a Way to Reach Sustainable Mezcal Production." *International Journal of Food Science & Technology* 58 (12): 6674–88. https://doi.org/10.1111/ijfs.16784.

Adame-Soto, P. Jaciel, Elva T. Aréchiga-Carvajal, Silvia M. González-Herrera, Martha R. Moreno-Jiménez, and Olga M. Rutiaga-Quiñones. 2023. "Characterization of Mating Type on Aroma Production and Metabolic Properties Wild *Kluyveromyces marxianus* Yeasts." *World Journal of Microbiology and Biotechnology* 39 (8): 216. https://doi.org/10.1007/s11274-023-03659-4.

Aguirre, X., C. Illsley, and J. Larson. 2006. "Dulce Semblanza de Los Mezcales Del Altiplano y Del Balsas." *México Desconocido* 352: 36-45.

Alcázar-Valle, Elba Montserrat. 2016. "Caracterización de Saponinas de *Agave durangensis* y *salmiana*, y su Efecto en la Pared y Membrana Celular de *Kluyveromyces marxianus* y *Saccharomyces cerevisiae*." Doctoral Dissertation, Mexico: Centro de Investigación y Asistencia en Tecnología y Diseño del Estado de Jalisco, A.C. https://ciatej.repositorioinstitucional.mx/jspui/bitstream/1023/421/1/Elba%20Montserrat%20Alc%c3%a1zar%20Valle.pdf.

Alcazar-Valle, Montserrat, Anne Gschaedler, Humberto Gutierrez-Pulido, Alejandro Arana-Sanchez, and Melchor Arellano-Plaza. 2019. "Fermentative Capabilities of Native Yeast Strains Grown on Juices from Different *Agave* Species Used for Tequila and Mezcal Production." *Brazilian Journal of Microbiology* 50 (2): 379–88. https://doi.org/10.1007/s42770-019-00049-7.

Aldrete-Tapia, A., R. Martínez-Peniche, D. Miranda-Castilleja, and M. Hernández-Iturriaga. 2018. "*Saccharomyces cerevisiae* Associated with the Spontaneous Fermentation of Tequila Agave Juice." *Journal of the Institute of Brewing. Institute of Brewing* 124 (3): 284–90. https://doi.org/10.1002/jib.499.

Aldrete-Tapia, J. A., P. Escalante-Minakata, R. A. Martínez-Peniche, M. L. Tamplin, and M. Hernández-Iturriaga. 2020. "Yeast and Bacterial Diversity, Dynamics and Fermentative Kinetics during Small-Scale Tequila Spontaneous Fermentation." *Food Microbiology* 86 (April): 103339. https://doi.org/10.1016/j.fm.2019.103339.

Álvarez-Ainza, M., M. Arellano-Plaza, F. J. De la Torre-González, J. Gallardo-Valdez, S. E. García-Barrón, A. García-Galaz, A. Gschaedler-Mathis, et al. 2017. "3. Bebidas Tradicionales, Nuevas y Alternativas. Sección II. Bebidas Destiladas de Agave." In *Panorama Del Aprovechamiento de Los Agaves En México*, edited by Anne Christine Gschaedler Mathis, Sandra Villarreal Hernández, Antonia Gutiérrez-Mora, Rosa Isela Ortiz Basurto, Casildo Rubén Moreno-Terrazas, Patricia Ester Lappe Oliveras, Claudia Patricia Larralde Corona, Silvia Maribel Contreras-Ramos, Gustavo Dávila Vázquez, and Juan Gallardo Valdéz, 165–214. CONACYT, CIATEJ, AGARED. http://ciatej.repositorioinstitucional.mx/jspui/handle/1023/646.

Álvarez-Ainza, M. L., K. A. Zamora-Quiñonez, G. M. Moreno-Ibarra, and E. Acedo-Félix. 2015. "Genomic Diversity of *Saccharomyces cerevisiae* Yeasts Associated with Alcoholic Fermentation of Bacanora Produced by Artisanal Methods." *Applied Biochemistry and Biotechnology* 175 (5): 2668–76. https://doi.org/10.1007/s12010-014-1469-y.

Alvarez-Ainza, Maritza Lizeth, Alfonso García-Galaz, Humberto Gonzalez-Rios, Mayra De la Torre-Martinez, Karina Alejandra Zamora-Quiñones, and Evelia Acedo-Félix. 2021. "Characterization and Selection of Native Yeast Isolated from Natural Fermentation for the Production of the Artisanal Beverage Bacanora." *Biotecnia* 23 (1): 21–27. https://doi.org/10.18633/biotecnia.v23i1.1221.

Álvarez-Ríos, Gonzalo D., Carmen Julia Figueredo-Urbina, and Alejandro Casas. 2020. “Physical, Chemical, and Microbiological Characteristics of Pulque: Management of a Fermented Beverage in Michoacán, Mexico.” *Foods (Basel, Switzerland)* 9 (3):361 https://doi.org/10.3390/foods9030361.

Álvarez-Ríos, Gonzalo D., Fernando Pacheco-Torres, Carmen J. Figueredo-Urbina, and Alejandro Casas. 2020. “Management, Morphological and Genetic Diversity of Domesticated Agaves in Michoacán, México.” *Journal of Ethnobiology and Ethnomedicine* 16 (1): 3. https://doi.org/10.1186/s13002-020-0353-9.

Amaya-Delgado, L., E. J. Herrera-López, Javier Arrizon, M. Arellano-Plaza, and A. Gschaedler. 2013. “Performance Evaluation of *Pichia kluyveri, Kluyveromyces marxianus* and *Saccharomyces cerevisiae* in Industrial Tequila Fermentation.” *World Journal of Microbiology and Biotechnology* 29 (5): 875–81. https://doi.org/10.1007/s11274-012-1242-8.

Arellano-Plaza, Melchor, Jesús Bernardo Paez-Lerma, Nicolás Oscar Soto-Cruz, Manuel R. Kirchmayr, and Anne Gschaedler Mathis. 2022. “Mezcal Production in Mexico: Between Tradition and Commercial Exploitation.” *Frontiers in Sustainable Food Systems* 6 (March): 832532. https://doi.org/10.3389/fsufs.2022.832532.

Arias-García, José Armando. 2008. “Diversidad Genética En Las Especies Del Complejo *Saccharomyces sensu stricto* de Fermentaciones Tradicionales.” Doctoral Dissertation, Spain: Facultad de Ciencias Biológicas, Universitat de Valencia. https://roderic.uv.es/bitstreams/296d4224-f359-4a4d-a64f-00501d02b115/download

Arrizon, Javier, Concetta Fiore, Guillermina Acosta, Patrizia Romano, and Anne Gschaedler. 2006. “Fermentation Behaviour and Volatile Compound Production by Agave and Grape Must Yeasts in High Sugar *Agave tequilana* and Grape Must Fermentations.” *Antonie van Leeuwenhoek* 89 (1): 181–89. https://doi.org/10.1007/s10482-005-9022-1.

Arrizon, Javier, Sandrine Morel, Anne Gschaedler, and Pierre Monsan. 2012. “Fructanase and Fructosyltransferase Activity of Non-*Saccharomyces* Yeasts Isolated from Fermenting Musts of Mezcal.” *Bioresource Technology* 110 (C): 560–65. https://doi.org/10.1016/j.biortech.2012.01.112.

Astudillo-Melgar, Fernando, Georgina Hernández-Chávez, María Elena Rodríguez-Alegría, Francisco Bolívar, and Adelfo Escalante. 2023. “Analysis of the Microbial Diversity and Population Dynamics during the Pulque Fermentation Process.” *Fermentation* 9 (4): 342. https://doi.org/10.3390/fermentation9040342.

Avelar-Rivas, J. Abraham, Iván Sedeño, Luis Fernando García-Ortega, Jose A. Urban Aragon, Eugenio Mancera, Alexander DeLuna, and Lucía Morales. 2024. “Recurrent Introgression and Geographical Stratification Shape *Saccharomyces cerevisiae* in the Neotropics.” *BioRxiv*. doi: https://doi.org/10.1101/2024.09.27.615306.

Bokulich, Nicholas A., Moe Ohta, Morgan Lee, and David A. Mills. 2014. “Indigenous Bacteria and Fungi Drive Traditional Kimoto Sake Fermentations.” *Applied and Environmental Microbiology* 80 (17): 5522–29. https://doi.org/10.1128/AEM.00663-14.

Bruman, Henry J. 2000. *Alcohol in Ancient Mexico*. Salt Lake City: University of Utah Press. https://archive.org/details/alcoholinancient00brum/page/n7/mode/2up.

Cabrera-Toledo, Dánae, Ofelia Vargas-Ponce, Sabina Ascencio-Ramírez, Luis Mario Valadez-Sandoval, Jessica Pérez-Alquicira, Judith Morales-Saavedra, and Oassis F. Huerta-Galván. 2020. “Morphological and Genetic Variation in Monocultures, Forestry Systems and Wild Populations of *Agave maximiliana* of Western Mexico: Implications for Its Conservation.” *Frontiers in Plant Science* 11 (June): 817. https://doi.org/10.3389/fpls.2020.00817.

Canonico, Laura, Francesca Comitini, and Maurizio Ciani. 2017. “*Torulaspora delbrueckii* Contribution in Mixed Brewing Fermentations with Different *Saccharomyces cerevisiae*

Strains." *International Journal of Food Microbiology* 259 (October): 7–13. https://doi.org/10.1016/j.ijfoodmicro.2017.07.017.

Cedeño-Cruz, Miguel. 2003. "Tequila Production from Agave: Historical Influences and Contemporary Processes." In *The Alcohol Textbook: A Reference for the Beverage, Fuel and Industrial Alcohol Industries*, edited by Kathryn Ann Jacques, T. Pearse Lyons, and Dave R. Kelsall, 223–45. Nottingham University Press.

Chacón-Vargas, Katherine, Julian Torres, Martha Giles-Gómez, Adelfo Escalante, and John G. Gibbons. 2020. "Genomic Profiling of Bacterial and Fungal Communities and Their Predictive Functionality during Pulque Fermentation by Whole-Genome Shotgun Sequencing." *Scientific Reports* 10 (1): 15115. https://doi.org/10.1038/s41598-020-71864-4.

Chai, Chunyue, Dan Lu, Jinli Liu, Eentao Wang, Xuemei Han, and Fengli Hui. 2024. "Three Novel Ascomycota (Saccharomycetes, Saccharomycetales) Yeast Species Derived from the Traditional Mexican Alcoholic Beverage Pulque." *MycoKeys* 109 (October): 187–206. https://doi.org/10.3897/mycokeys.109.123870.

Colunga-GarcíaMarín, Patricia, Ignacio Torres-García, Alejandro Casas, Carmen J. Figueredo-Urbina, Selene Rangel-Landa, América Delgado-Lemus, Ofelia Vargas, et al. 2017. "Los Agaves y Las Prácticas Mesoamericanas de Aprovechamiento, Manejo y Domesticación." In *Domesticación En El Continente Americano*, edited by Alejandro Casas, Juan Torres-Guevara, and Fabiola Parra-Rondinel, 2:273–308. Universidad Nacional Autónoma de México.

Colunga-GarcíaMarín, Patricia, and Daniel Zizumbo-Villarreal. 2007. "Tequila and Other *Agave* Spirits from West-Central Mexico: Current Germplasm Diversity, Conservation and Origin." *Biodiversity and Conservation* 16: 1653–67. https://doi.org/10.1007/978-1-4020-6444-9_6.

CONABIO. 2006. "Mezcales y Diversidad." Comisión Nacional para el Conocimiento y Uso de la Biodiversidad. México. https://bioteca.biodiversidad.gob.mx/janium-bin/janium_zui.pl?jzd=/janium/Documentos/ETAPA06/AP/5324/Mezcales.jzd&fn=5324.

CONABIO. 2009. "Ecosistemas de México." Biodiversidad Mexicana. 2009. https://www.biodiversidad.gob.mx/ecosistemas/ecosismex.

De la Torre-González, Francisco Javier, José Alberto Narváez-Zapata, Víctor Eric López-y-López, and Claudia Patricia Larralde-Corona. 2016. "Ethanol Tolerance Is Decreased by Fructose in *Saccharomyces* and non-*Saccharomyces* Yeasts." *LWT--Food Science and Technology* 67 (April): 1–7. https://doi.org/10.1016/j.lwt.2015.11.024.

De Vuyst, Luc, and Frédéric Leroy. 2020. "Functional Role of Yeasts, Lactic Acid Bacteria and Acetic Acid Bacteria in Cocoa Fermentation Processes." *FEMS Microbiology Reviews* 44 (4): 432–53. https://doi.org/10.1093/femsre/fuaa014.

Díaz-Montaño, Dulce M., Marie-Line Délia, Mirna Estarrón-Espinosa, and Pierre Strehaiano. 2008. "Fermentative Capability and Aroma Compound Production by Yeast Strains Isolated from *Agave tequilana* Weber Juice." *Enzyme and Microbial Technology* 42 (7): 608–16. https://doi.org/10.1016/j.enzmictec.2007.12.007.

Dzialo, Maria C., Rahel Park, Jan Steensels, Bart Lievens, and Kevin J. Verstrepen. 2017. "Physiology, Ecology and Industrial Applications of Aroma Formation in Yeast." *FEMS Microbiology Reviews* 41 (Supp_1): S95–128. https://doi.org/10.1093/femsre/fux031.

Enríquez-Salazar, M. Isabel, Fabiola Veana, Cristóbal N. Aguilar, Iliana M. De la Garza-Rodríguez, Mercedes G. López, Olga M. Rutiaga-Quiñones, Jesús A. Morlett-Chávez, and Raúl Rodríguez-Herrera. 2017. "Microbial Diversity and Biochemical Profile of Aguamiel Collected from *Agave salmiana* and *A. atrovirens* during Different Seasons of Year." *Food Science and Biotechnology* 26 (4): 1003–11. https://doi.org/10.1007/s10068-017-0141-z.

Escalante, Adelfo, Martha Giles-Gómez, Georgina Hernández, María Soledad Córdova-Aguilar, Agustín López-Munguía, Guillermo Gosset, and Francisco Bolívar. 2008. "Analysis of

Bacterial Community during the Fermentation of Pulque, a Traditional Mexican Alcoholic Beverage, Using a Polyphasic Approach.” *International Journal of Food Microbiology* 124 (2): 126–34. https://doi.org/10.1016/j.ijfoodmicro.2008.03.003.

Escalante, Adelfo, David R. López-Soto, Judith E. Velázquez-Gutiérrez, Martha Giles-Gómez, Francisco Bolívar, and Agustín López-Munguía. 2016. “Pulque, a Traditional Mexican Alcoholic Fermented Beverage: Historical, Microbiological, and Technical Aspects.” *Frontiers in Microbiology* 7 (June): 1026. https://doi.org/10.3389/fmicb.2016.01026.

Escalante, Adelfo, María Elena Rodríguez, Alfredo Martínez, Agustín López-Munguía, Francisco Bolívar, and Guillermo Gosset. 2004. “Characterization of Bacterial Diversity in Pulque, a Traditional Mexican Alcoholic Fermented Beverage, as Determined by 16S RDNA Analysis.” *FEMS Microbiology Letters* 235 (2): 273–79. https://doi.org/10.1016/j.femsle.2004.04.045.

Escalante-Minakata, P., H. P. Blaschek, A. P. Barba de la Rosa, L. Santos, and A. De León-Rodríguez. 2008. “Identification of Yeast and Bacteria Involved in the Mezcal Fermentation of *Agave salmiana*.” *Letters in Applied Microbiology* 46 (6): 626–30. https://doi.org/10.1111/j.1472-765X.2008.02359.x.

Espinosa Paz, Horacio, Ernesto Bravo Mosqueda, Porfirio López López, and Carlos Arredondo Velásquez. 2005. “El Agave Mezcalero de Oaxaca. Avances de Investigación.” Libro Técnico No. 3. México: Campo Experimental Valles Centrales de Oaxaca, CIRPAS, INIFAP Oaxaca. https://biblat.unam.mx/es/revista/ciencia-forestal-en-mexico/articulo/espinosa-h-bravo-e-lopez-p-arredondo-c-el-agave-mezcalero-de-oaxaca-avances-de-investigacion-libro-tecnico-no-3-mexico-campo-experimental-valles-centrales-de-oaxaca-cirpas-inifap-oaxaca-2005-180-p-isbn-9704300050

Espinoza-Martinez, Victor Adrian, Peggy Elizabeth Alvarez-Gutierrez, Felipe de Jesus Palma-Cruz, Raul Enriquez-Valencia, Marcos Pedro Ramirez-Lopez, Claudia Lopez-Sanchez, and Hector Gilberto Vazquez-Lopez. 2023. “Influence of the Biotechnological Process of Mezcal Fermentation on Yeast Diversity in Four *palenques* of Oaxaca, Mexico.” *Beverages* 9 (4): 99. https://doi.org/10.3390/beverages9040099.

Fernandes, Ticiana, Flávia Silva-Sousa, Fábio Pereira, Teresa Rito, Pedro Soares, Ricardo Franco-Duarte, and Maria João Sousa. 2021. “Biotechnological Importance of *Torulaspora delbrueckii*: From the Obscurity to the Spotlight.” *Journal of Fungi (Basel, Switzerland)* 7 (9): 712. https://doi.org/10.3390/jof7090712.

Fiore, Concetta, Javier Arrizon, Anne Gschaedler, Javier Flores, and Patrizia Romano. 2005. “Comparison between Yeasts from Grape and Agave Musts for Traits of Technological Interest.” *World Journal of Microbiology and Biotechnology* 21 (6–7): 1141–47. https://doi.org/10.1007/s11274-005-0196-5.

Gallegos-Casillas, Porfirio, Luis F. García-Ortega, Adriana Espinosa-Cantú, J. Abraham Avelar-Rivas, Carolina G. Torres-Lagunes, Adrián Cano-Ricardez, Ángela M. García-Acero, et al. 2024. “Yeast Diversity in Open Agave Fermentations across Mexico.” *Yeast (Chichester, England)* 41 (1–2): 35–51. https://doi.org/10.1002/yea.3913.

García Mendoza, Abisaí. 2002. “Distribution of *Agave* (Agavaceae) in Mexico.” *Cactus and Succulent Journal* 74 (4): 177–87.

García-Ortega, Luis F., Maritrini Colón-González, Iván Sedeño, Erick Santiago-Garduño, J. Abraham Avelar-Rivas, Manuel R. Kirchmayr, Alexander DeLuna, Luis Delaye, Lucia Morales, and Eugenio Mancera. 2022. “Draft Genome Sequence of a *Kazachstania humilis* Strain Isolated from Agave Fermentation.” *Microbiology Resource Announcements* 11 (3): e0115421. https://doi.org/10.1128/mra.01154-21.

Garibay-Marcelo, María de Los Ángeles. 2019. “Caracterización de La Diversidad de Levaduras Presentes Durante El Proceso de Fermentación Del Mezcal En El Estado de Guerrero.” BSc Dissertation, Universidad Autónoma de Guerrero.

Gentry, Howard Scott. 1982. *Agaves of Continental North America*. University of Arizona Press. https://doi.org/10.2307/j.ctv1t4m2h4.

Gómez-Márquez, Carolina, Dania Sandoval-Nuñez, Anne Gschaedler, Teresa Romero-Gutiérrez, Lorena Amaya-Delgado, and J. Alejandro Morales. 2021. "Diploid Genome Assembly of *Kluyveromyces marxianus* NRRL Y-50883 (SLP1)." *G3: Genes|Genomes|Genetics* 12 (October). https://doi.org/10.1093/g3journal/jkab347.

González-Robles, Ivonne Wendolyne, Mirna Estarrón-Espinosa, and Dulce María Díaz-Montaño. 2015. "Fermentative Capabilities and Volatile Compounds Produced by *Kloeckera*/*Hanseniaspora* and *Saccharomyces* Yeast Strains in Pure and Mixed Cultures during *Agave tequilana* Juice Fermentation." *Antonie van Leeuwenhoek* 108 (3): 525–36. https://doi.org/10.1007/s10482-015-0506-3.

Gschaedler, Anne Christine, Antonia Gutiérrez-Mora, Silvia Maribel Contreras-Ramos, Gustavo Dávila-Vazquez, and Juan Gallardo-Valdez. 2017. *Panorama Del Aprovechamiento de Los Agaves En México*. CONACYT, CIATEJ, AGARED. http://ciatej.repositorioinstitucional.mx/jspui/handle/1023/646.

Gutiérrez-Coronado, M. L., E. Acedo-Félix, and A. I. Valenzuela-Quintanar. 2007. "Industria Del Bacanora y Su Proceso de Elaboración / Bacanora Industry and Its Process of Production." *Ciencia y Tecnologia Alimentaria* 5 (5): 394–404. https://doi.org/10.1080/11358120709487718.

Gutiérrez-Gamboa, G., J. Portu, P. Santamaría, R. López, and T. Garde-Cerdán. 2017. "Effects on Grape Amino Acid Concentration through Foliar Application of Three Different Elicitors." *Food Research International* 99 (September): 688–92. https://doi.org/10.1016/j.foodres.2017.06.022.

Han, Da-Yong, Pei-Jie Han, Karl Rumbold, Anbessa Dabassa Koricha, Shou-Fu Duan, Liang Song, Jun-Yan Shi, Kuan Li, Qi-Ming Wang, and Feng-Yan Bai. 2021. "Adaptive Gene Content and Allele Distribution Variations in the Wild and Domesticated Populations of *Saccharomyces cerevisiae*." *Frontiers in Microbiology* 12 (February): 631250. https://doi.org/10.3389/fmicb.2021.631250.

Hernández-Cortés, Guillermo, Juan Octavio Valle-Rodríguez, Enrique J. Herrera-López, Dulce María Díaz-Montaño, Yolanda González-García, Héctor B. Escalona-Buendía, and Jesús Córdova. 2016. "Improvement on the Productivity of Continuous Tequila Fermentation by *Saccharomyces cerevisiae* of *Agave tequilana* Juice with Supplementation of Yeast Extract and Aeration." *AMB Express* 6 (1): 47. https://doi.org/10.1186/s13568-016-0218-8.

INEGI. 2003. "Conjunto de Datos Vectoriales de La Carta de Vegetación Primaria." Aguascalientes, Ags., Mexico: Instituto Nacional de Estadística, Geografía e Informática (INEGI). https://datos.gob.mx/busca/dataset/conjunto-de-datos-vectoriales-de-la-carta-de-vegetacion-primaria-escala-1- 1-000-000-nivel-i-y-i/resource/f8159fc8-4ddd-4d6e-ab9d-198743f5d3ee

Iwaki, Aya, Shinsuke Ohnuki, Yohei Suga, Shingo Izawa, and Yoshikazu Ohya. 2013. "Vanillin Inhibits Translation and Induces Messenger Ribonucleoprotein (MRNP) Granule Formation in *Saccharomyces cerevisiae*: Application and Validation of High-Content, Image-Based Profiling." *PloS One* 8 (4): e61748. https://doi.org/10.1371/journal.pone.0061748.

Jacques-Hernandez, Cuauhtemoc, Octavio Herrera-Perez, and José Ramírez de León. 2007. "El Maguey Mezcalero y La Agroindustria Del Mezcal En Tamaulipas." In *En lo ancestral hay futuro: del tequila, los mezcales y otros agaves*, edited by P. C. García Marín, A. Larqué Saavedra, L. E. Eguiarte and D. Zizumbo-Villarreal, 287-317, Mexico: CICY-CONACYT-CONABIO-INE.

Jara-Servin, Angélica, Luis D. Alcaraz, Sabino I. Juarez-Serrano, Aarón Espinosa-Jaime, Ivan Barajas, Lucia Morales, Alexander DeLuna, Antonio Hernández-López, and Eugenio Mancera. 2025. "Microbial Communities Thriving in Agave Fermentations Are Locally

Influenced across Diverse Biogeographic Regions." *Environmental Microbiology Reports* 17 (1): e70057. https://doi.org/10.1111/1758-2229.70057.

Kirchmayr, Manuel R., Luis E. Segura-García, Patricia Lappe-Oliveras, Rubén Moreno-Terrazas, Mayela de la Rosa, and Anne Gschaedler-Mathis. 2017. "Impact of Environmental Conditions and Process Modifications on Microbial Diversity, Fermentation Efficiency and Chemical Profile during the Fermentation of *Mezcal* in Oaxaca." *LWT--Food Science and Technology* 79 (June): 160–69. https://doi.org/10.1016/j.lwt.2016.12.052.

Kowallik, Vienna, and Duncan Greig. 2016. "A Systematic Forest Survey Showing an Association of *Saccharomyces paradoxus* with Oak Leaf Litter." *Environmental Microbiology Reports* 8 (5): 833–41. https://doi.org/10.1111/1758-2229.12446.

Kurtzman, Cletus P. 2011. "Chapter 75 - Torulaspora Lindner (1904)." In *The Yeasts (Fifth Edition)*, edited by Cletus P. Kurtzman, Jack W. Fell, and Teun Boekhout, 867–74. London: Elsevier. https://doi.org/10.1016/B978-0-444-52149-1.00075-6.

Lachance, Marc-André. 1995. "Yeast Communities in a Natural Tequila Fermentation." *Antonie van Leeuwenhoek* 68 (2): 151–60. https://doi.org/10.1007/bf00873100.

Lane, Melanie M., and John P. Morrissey. 2010. "*Kluyveromyces marxianus*: A Yeast Emerging from Its Sister's Shadow." *Fungal Biology Reviews* 24 (1–2): 17–26. https://doi.org/10.1016/j.fbr.2010.01.001.

Lappe-Oliveras, Patricia, Morena Avitia, Sara Darinka Sánchez-Robledo, Ana Karina Castillo-Plata, Lorena Pedraza, Guillermo Baquerizo, and Sylvie Le Borgne. 2023. "Genotypic and Phenotypic Diversity of *Kluyveromyces marxianus* Isolates Obtained from the Elaboration Process of Two Traditional Mexican Alcoholic Beverages Derived from Agave: Pulque and Henequen (*Agave fourcroydes*) Mezcal." *Journal of Fungi (Basel, Switzerland)* 9 (8):795. https://doi.org/10.3390/jof9080795.

Lappe-Oliveras, Patricia, Rubén Moreno-Terrazas, Javier Arrizón-Gaviño, Teófilo Herrera-Suárez, Abisaí García-Mendoza, and Anne Gschaedler-Mathis. 2008. "Yeasts Associated with the Production of Mexican Alcoholic Nondistilled and Distilled *Agave* Beverages." *FEMS Yeast Research* 8 (7): 1037–52. https://doi.org/10.1111/j.1567-1364.2008.00430.x.

Larralde-Corona, Claudia Patricia, Francisco Javier De la Torre-González, Pedro Alberto Vázquez-Landaverde, Dittmar Hahn, and José Alberto Narváez-Zapata. 2021. "Rational Selection of Mixed Yeasts Starters for *Agave* Must Fermentation." *Frontiers in Sustainable Food Systems* 5. https://doi.org/10.3389/fsufs.2021.684228.

León-Rodríguez, Antonio, Pilar Escalante-Minakata, María I. Jiménez-García, Leandro G. Ordoñez-Acevedo, José L. Flores-Flores, and Ana P. Barba de la Rosa. 2008. "Characterization of Volatile Compounds from Ethnic *Agave* Alcoholic Beverages by Gas Chromatography-Mass Spectrometry." *Food Technology and Biotechnology* 46 (4): 448–55. https://www.semanticscholar.org/paper/fe183e3639dd128ea15ec410d2ffeed28fc5c78d.

Liu, Pei-Tong, Lin Lu, Chang-Qing Duan, and Guo-Liang Yan. 2016. "The Contribution of Indigenous Non-*Saccharomyces* Wine Yeast to Improved Aromatic Quality of Cabernet Sauvignon Wines by Spontaneous Fermentation." *LWT--Food Science and Technology* 71: 356–63. https://doi.org/10.1016/j.lwt.2016.04.031.

López-Alvarez, Arnoldo, Alma Laura Díaz-Pérez, Carlos Sosa-Aguirre, Lourdes Macías-Rodríguez, and Jesús Campos-García. 2012. "Ethanol Yield and Volatile Compound Content in Fermentation of Agave Must by *Kluyveromyces marxianus* UMPe-1 Comparing with *Saccharomyces cerevisiae* Baker's Yeast Used in Tequila Production." *Journal of Bioscience and Bioengineering* 113 (5): 614–18. https://doi.org/10.1016/j.jbiosc.2011.12.015.

López-Ramírez, J. E., S. T. Martín-del-Campo, H. Escalona-Buendía, J. A. García-Fajardo, and M. Estarrón-Espinosa. 2013. "Physicochemical Quality of Tequila during Barrel

Maturation. A Preliminary Study." *CyTA - Journal of Food* 11 (3): 223–33. https://doi.org/10.1080/19476337.2012.727033.

Lozano-Aguirre, Luis, Morena Avitia, Patricia Lappe-Oliveras, Cuauhtémoc Licona-Cassani, Miguel A. Cevallos, and Sylvie Le Borgne. 2024. "Draft Genomes of Four *Kluyveromyces marxianus* Isolates Retrieved from the Elaboration Process of Henequen (*Agave fourcroydes*) Mezcal." *Microbiology Resource Announcements* 13 (3): e0086123. https://doi.org/10.1128/mra.00861-23.

MacNeish, Richard S., and Douglas S. Byers. 1967. *The Prehistory of the Tehuacan Valley*. Edited by Douglas S. Byers. Vol. 1, Enironment and subsistence. Univ. Texas Press.

Madden, Anne A., Mary Jane Epps, Tadashi Fukami, Rebecca E. Irwin, John Sheppard, D. Magdalena Sorger, and Robert R. Dunn. 2018. "The Ecology of Insect-Yeast Relationships and Its Relevance to Human Industry." *Proceedings. Biological Sciences* 285 (1875). https://doi.org/10.1098/rspb.2017.2733.

Mancilla-Margalli, N. Alejandra, and Mercedes G. López. 2006. "Water-Soluble Carbohydrates and Fructan Structure Patterns from *Agave* and *Dasylirion* Species." *Journal of Agricultural and Food Chemistry* 54 (20): 7832–39. https://doi.org/10.1021/jf060354v.

Mancilla-Margalli, Norma A., and Mercedes G. López. 2002. "Generation of Maillard Compounds from Inulin during the Thermal Processing of *Agave tequilana* Weber Var. Azul." *Journal of Agricultural and Food Chemistry* 50 (4): 806–12. https://doi.org/10.1021/jf0110295.

Martínez-Estrada, Sandra, José Narváez-Zapata, Raul Rodriguez, Julio Grijalva-Ávila, José Gurrola-Reyes, Claudia Patricia Larralde-Corona, and Isaías Hernández. 2024. "Diversity of Culturable Yeasts Associated with the Technification Level in the Process of Mezcal Production in the State of Durango." *Fermentation* 10 (March): 147. https://doi.org/10.3390/fermentation10030147.

Méndez-Zamora, Andrés, Daniel Oswaldo Gutiérrez-Avendaño, Melchor Arellano-Plaza, Francisco Javier De la Torre González, Iliana Barrera-Martínez, Anne Gschaedler Mathis, and Leticia Casas-Godoy. 2021. "The Non-*Saccharomyces* Yeast *Pichia kluyveri* for the Production of Aromatic Volatile Compounds in Alcoholic Fermentation." *FEMS Yeast Research* 20 (8). https://doi.org/10.1093/femsyr/foaa067.

Meriggi, Niccolo, Monica Di Paola, Duccio Cavalieri, and Irene Stefanini. 2020. "*Saccharomyces cerevisiae* - Insects Association: Impacts, Biogeography, and Extent." *Frontiers in Microbiology* 11 (July): 1629. https://doi.org/10.3389/fmicb.2020.01629.

Molina-Guerrero, J. A., J. E. Botello-Álvarez, A. Estrada-Baltazar, J. L. Navarrete-Bolaños, H. Jiménez-Islas, M. Cárdenas-Manríquez, and R. Rico-Martínez. 2007. "Compuestos Volátiles En El Mezcal." *Revista Mexicana de Ingeniería Química* 6 (1): 41–50. https://www.redalyc.org/pdf/620/62060106.pdf.

Mora-López, J. Luis, J. Antonio Reyes-Agüero, J. Luis Flores-Flores, C. Beatriz Peña-Valdivia, and J. Rogelio Aguirre-Rivera. 2011. "Morphological Variation and Humanization of *Agave* Genus, Salmianae Section." *Agrociencia*. 45 (4):465. https://www.cabdirect.org/cabdirect/abstract/20113258028.

Narváez-Zapata, J. A., R. A. Rojas-Herrera, I. C. Rodríguez-Luna, and C. P. Larralde-Corona. 2010. "Culture-Independent Analysis of Lactic Acid Bacteria Diversity Associated with Mezcal Fermentation." *Current Microbiology* 61 (5): 444–50. https://doi.org/10.1007/s00284-010-9636-z.

Narvhus, Judith A., and Tendekayi Henry Gadaga. 2003. "The Role of Interaction between Yeasts and Lactic Acid Bacteria in African Fermented Milks: A Review." *International Journal of Food Microbiology* 86 (1–2): 51–60. https://doi.org/10.1016/s0168-1605(03)00247-2.

Navarrete-Bolaños, J. L., and O. Serrato-Joya. 2023. "A Novel Strategy to Construct Multi-Strain Starter Cultures: An Insight to Evolve from Natural to Directed Fermentation." *Preparative Biochemistry & Biotechnology* 53 (10): 1199–1209. https://doi.org/10.1080/10826068.2023.2177870.

Nolasco-Cancino, Hipócrates, Jorge A. Santiago-Urbina, Carmen Wacher, and Francisco Ruíz-Terán. 2018. "Predominant Yeasts during Artisanal Mezcal Fermentation and Their Capacity to Ferment Maguey Juice." *Frontiers in Microbiology* 9 (December): 2900. https://doi.org/10.3389/fmicb.2018.02900.

Nuñez-Guerrero, Martha E., Elizabeth Salazar-Vázquez, Jesús B. Páez-Lerma, Raúl Rodríguez-Herrera, and Nicolás O. Soto-Cruz. 2019. "Physiological Characterization of Two Native Yeasts in Pure and Mixed Culture Using Fermentations of Agave Juice." *Ciencia e Investigación Agraria* 46 (1): 1–11. https://doi.org/10.7764/rcia.v46i1.1880.

Nuñez-Guerrero, Martha Eugenia, Jesús Bernardo Páez-Lerma, Olga Miriam Rutiaga-Quiñones, Silvia Marina González-Herrera, and Nicolás Oscar Soto-Cruz. 2016. "Performance of Mixtures of *Saccharomyces* and Non-*Saccharomyces* Native Yeasts during Alcoholic Fermentation of *Agave duranguensis* Juice." *Food Microbiology* 54 (April): 91–97. https://doi.org/10.1016/j.fm.2015.10.011.

O'Donnell, Samuel, Jia-Xing Yue, Omar Abou Saada, Nicolas Agier, Claudia Caradec, Thomas Cokelaer, Matteo De Chiara, et al. 2023. "Telomere-to-Telomere Assemblies of 142 Strains Characterize the Genome Structural Landscape in *Saccharomyces cerevisiae*." *Nature Genetics* 55 (8): 1390–99. https://doi.org/10.1038/s41588-023-01459-y.

Ojeda-Linares, César, Gonzalo D. Álvarez-Ríos, Carmen Julia Figueredo-Urbina, Luis Alfredo Islas, Patricia Lappe-Oliveras, Gary Paul Nabhan, Ignacio Torres-García, Mariana Vallejo, and Alejandro Casas. 2021. "Traditional Fermented Beverages of Mexico: A Biocultural Unseen Foodscape." *Foods (Basel, Switzerland)* 10 (10): 2390. https://doi.org/10.3390/foods10102390.

Ortiz-Basurto, Rosa Isela, Gérald Pourcelly, Thierry Doco, Pascale Williams, Manuel Dornier, and Marie-Pierre Belleville. 2008. "Analysis of the Main Components of the Aguamiel Produced by the Maguey-Pulquero (*Agave mapisaga*) throughout the Harvest Period." *Journal of Agricultural and Food Chemistry* 56 (10): 3682–87. https://doi.org/10.1021/jf072767h.

Ortiz-Merino, Raúl A., Javier A. Varela, Aisling Y. Coughlan, Hisashi Hoshida, Wendel B. da Silveira, Caroline Wilde, Niels G. A. Kuijpers, Jan-Maarten Geertman, Kenneth H. Wolfe, and John P. Morrissey. 2018. "Ploidy Variation in *Kluyveromyces marxianus* Separates Dairy and Non-Dairy Isolates." *Frontiers in Genetics* 9 (March): 94. https://doi.org/10.3389/fgene.2018.00094.

Páez-Lerma, Jesús B., Armando Arias-García, Olga M. Rutiaga-Quiñones, Eladio Barrio, and Nicolás O. Soto-Cruz. 2013. "Yeasts Isolated from the Alcoholic Fermentation of *Agave duranguensis* During Mezcal Production." *Food Biotechnology* 27 (4): 342–56. https://doi.org/10.1080/08905436.2013.840788.

Peris, David, Emily J. Ubbelohde, Meihua Christina Kuang, Jacek Kominek, Quinn K. Langdon, Marie Adams, Justin A. Koshalek, et al. 2023. "Macroevolutionary Diversity of Traits and Genomes in the Model Yeast Genus *Saccharomyces*." *Nature Communications* 14 (1): 690. https://doi.org/10.1038/s41467-023-36139-2.

Peter, Jackson, Matteo De Chiara, Anne Friedrich, Jia-Xing Yue, David Pflieger, Anders Bergström, Anastasie Sigwalt, et al. 2018. "Genome Evolution across 1,011 *Saccharomyces cerevisiae* Isolates." *Nature* 556 (7701): 339–44. https://doi.org/10.1038/s41586-018-0030-5.

Pinal, L., E. Cornejo, M. Arellano, E. Herrera, L. Nuñez, J. Arrizon, and A. Gschaedler. 2009. "Effect of *Agave tequilana* Age, Cultivation Field Location and Yeast Strain on Tequila Fermentation Process." *Journal of Industrial Microbiology & Biotechnology* 36 (5): 655–61. https://doi.org/10.1007/s10295-009-0534-y.

Pontes, Ana, Mathias Hutzler, Patrícia H. Brito, and José Paulo Sampaio. 2020. "Revisiting the Taxonomic Synonyms and Populations of *Saccharomyces cerevisiae*-Phylogeny,

Phenotypes, Ecology and Domestication.” *Microorganisms* 8 (6): 903. https://doi.org/10.3390/microorganisms8060903.

Ramirez-Córdova, Jesús, Jenny Drnevich, Jaime Alberto Madrigal-Pulido, Javier Arrizon, Kirk Allen, Moisés Martínez-Velázquez, and Ikuri Alvarez-Maya. 2012. “Transcriptome Analysis Identifies Genes Involved in Ethanol Response of *Saccharomyces cerevisiae* in *Agave tequilana* Juice.” *Antonie van Leeuwenhoek* 102 (2): 247–55. https://doi.org/10.1007/s10482-012-9733-z.

Ramírez-Guzmán, K. Nathiely, Cristian Torres-León, Gloria A. Martinez-Medina, Orlando de la Rosa, Ayerim Hernández-Almanza, O. Berenice Alvarez-Perez, Rafael Araujo, et al. 2019. “15 - Traditional Fermented Beverages in Mexico.” In *Fermented Beverages*, edited by Alexandru Mihai Grumezescu and Alina Maria Holban, 605–35. Woodhead Publishing. https://doi.org/10.1016/B978-0-12-815271-3.00015-4.

Rocha-Arriaga, Carolina, and Alfredo Cruz-Ramirez. 2022. “Yeast and Nonyeast Fungi: The Hidden Allies in Pulque Fermentation.” *Current Opinion in Food Science* 47 (100878): 100878. https://doi.org/10.1016/j.cofs.2022.100878.

Rocha-Arriaga, Carolina, Annie Espinal-Centeno, Shamayim Martinez-Sánchez, Juan Caballero-Pérez, Luis D. Alcaraz, and Alfredo Cruz-Ramírez. 2020. “Deep Microbial Community Profiling along the Fermentation Process of Pulque, a Biocultural Resource of Mexico.” *Microbiological Research* 241 (126593): 126593. https://doi.org/10.1016/j.micres.2020.126593.

Ruiz-Terán, Francisco, Paulina N. Martínez-Zepeda, Sara Y. Geyer-de la Merced, Hipócrates Nolasco-Cancino, and Jorge A. Santiago-Urbina. 2019. “Mezcal: Indigenous *Saccharomyces cerevisiae* Strains and Their Potential as Starter Cultures.” *Food Science and Biotechnology* 28 (2): 459–67. https://doi.org/10.1007/s10068-018-0490-2.

Sampaio, José Paulo, and Paula Gonçalves. 2008. “Natural Populations of *Saccharomyces kudriavzevii* in Portugal Are Associated with Oak Bark and Are Sympatric with *S. cerevisiae* and *S. paradoxus*.” *Applied and Environmental Microbiology* 74 (7): 2144–52. https://doi.org/10.1128/AEM.02396-07.

Sanchez-Marroquin, Alfredo, and P. H. Hope. 1953. “Agave Juice, Fermentation and Chemical Composition Studies of Some Species.” *Journal of Agricultural and Food Chemistry* 1 (3): 246–49. https://doi.org/10.1021/jf60003a007.

Schabort, Du Toit W. P., Precious K. Letebele, Laurinda Steyn, Stephanus G. Kilian, and James C. du Preez. 2016. “Differential RNA-Seq, Multi-Network Analysis and Metabolic Regulation Analysis of *Kluyveromyces marxianus* Reveals a Compartmentalised Response to Xylose.” *PloS One* 11 (6): e0156242. https://doi.org/10.1371/journal.pone.0156242.

Segura-García, Luis E., Patricia Taillandier, Cedric Brandam, and Anne Gschaedler. 2015. “Fermentative Capacity of *Saccharomyces* and Non-*Saccharomyces* in Agave Juice and Semi-Synthetic Medium.” *LWT - Food Science and Technology* 60 (1): 284–91. https://doi.org/10.1016/j.lwt.2014.08.005.

Serra-Puche, Mari Carmen, and Jesús Carlos Lazcano-Arce. 2016. *El Mezcal, Una Bebida Prehispánica: Estudios Etnoarqueológicos*. Universidad Nacional Autónoma de México, Instituto de Investigaciones Antropológicas.

Spurley, William J., Kaitlin J. Fisher, Quinn K. Langdon, Kelly V. Buh, Martin Jarzyna, Max A. B. Haase, Kayla Sylvester, et al. 2021. “Substrate, Temperature, and Geographical Patterns among Nearly 2000 Natural Yeast Isolates.” *Yeast* , November 39 (1-2): 55-68. https://doi.org/10.1002/yea.3679.

Tellini, Nicolò, Matteo De Chiara, Simone Mozzachiodi, Lorenzo Tattini, Chiara Vischioni, Elena S. Naumova, Jonas Warringer, Anders Bergström, and Gianni Liti. 2024. “Ancient and Recent Origins of Shared Polymorphisms in Yeast.” *Nature Ecology & Evolution* 8 (4): 761–76. https://doi.org/10.1038/s41559-024-02352-5.

Tello-Balderas, J. Jesús, and Edmundo García-Moya. 2017. "El Maguey (Agave, Subgénero Agave) En El Altiplano Potosino-Zacatecano." *Botanical Sciences*. https://doi.org/10.17129/botsci.1350.

Tofalo, Rosanna, Vincenzina Fusco, Christina Böhnlein, Jan Kabisch, Antonio F. Logrieco, Diana Habermann, Gyu-Sung Cho, et al. 2020. "The Life and Times of Yeasts in Traditional Food Fermentations." *Critical Reviews in Food Science and Nutrition* 60 (18): 3103–32. https://doi.org/10.1080/10408398.2019.1677553.

Tufariello, Maria, Mariagiovanna Fragasso, Joana Pico, Annarita Panighel, Simone Diego Castellarin, Riccardo Flamini, and Francesco Grieco. 2021. "Influence of Non-*Saccharomyces* on Wine Chemistry: A Focus on Aroma-Related Compounds." *Molecules (Basel, Switzerland)* 26 (3): 644. https://doi.org/10.3390/molecules26030644.

Tyakht, Alexander, Anna Kopeliovich, Natalia Klimenko, Daria Efimova, Nikita Dovidchenko, Vera Odintsova, Mikhail Kleimenov, et al. 2021. "Characteristics of Bacterial and Yeast Microbiomes in Spontaneous and Mixed-Fermentation Beer and Cider." *Food Microbiology* 94 (103658): 103658. https://doi.org/10.1016/j.fm.2020.103658.

Valencia-Avalos, Susana. 2010. "Notes on the Genus *Quercus* in Mexico." *International Oak Journal* 21 (2010): 100–120.

Valle-Rodríguez, Juan Octavio, Guillermo Hernández-Cortés, Jesús Córdova, Mirna Estarrón-Espinosa, and Dulce María Díaz-Montaño. 2012. "Fermentation of *Agave tequilana* Juice by *Kloeckera africana*: Influence of Amino-Acid Supplementations." *Antonie van Leeuwenhoek* 101 (2): 195–204. https://doi.org/10.1007/s10482-011-9622-x.

Vargas-Ponce, Ofelia, Daniel Zizumbo-Villarreal, Jaime Martínez-Castillo, Julián Coello-Coello, and Patricia Colunga-GarcíaMarín. 2009. "Diversity and Structure of Landraces of *Agave* Grown for Spirits under Traditional Agriculture: A Comparison with Wild Populations of *A. angustifolia* (Agavaceae) and Commercial Plantations of *A. tequilana*." *American Journal of Botany* 96 (2): 448–57. https://doi.org/10.3732/ajb.0800176.

Vázquez-Pérez, Nancy. 2015. "Variación Morfológica y Genética de *Agave karwinskii* (Agavaceae) En Los Estados de Oaxaca y Puebla." Master Dissertation, Universidad Nacional Autónoma de México. http://132.248.9.195/ptd2015/noviembre/0738078/Index.html.

Vera-Guzmán, Araceli, Rosa I. Guzmán-Gerónimo, and Mercedes G. López. 2010. "Major and Minor Compounds in a Mexican Spirit, Young Mezcal Coming from Two *Agave* Species." *Czech Journal of Food Sciences* 28 (2): 127–32. https://doi.org/10.17221/56/2009-cjfs.

Vera-Guzmán, Araceli, Rosa Guzmán-Gerónimo, Mercedes López, and José Chávez-Servia. 2018. "Volatile Compound Profiles in Mezcal Spirits as Influenced by *Agave* Species and Production Processes." *Beverages* 4 (1): 9. https://doi.org/10.3390/beverages4010009.

Vera-Guzmán, Araceli Minerva, Mercedes Guadalupe López, and José Luis Chávez-Servia. 2012. "Chemical Composition and Volatile Compounds in the Artisanal Fermentation of Mezcal in Oaxaca, Mexico." *African Journal of Biotechnology* 11 (78): 14344–53. https://doi.org/10.4314/ajb.v11i78.

Verdugo Valdez, A., L. Segura Garcia, M. Kirchmayr, P. Ramírez Rodríguez, A. González Esquinca, R. Coria, and A. Gschaedler Mathis. 2011. "Yeast Communities Associated with Artisanal Mezcal Fermentations from *Agave salmiana*." *Antonie van Leeuwenhoek* 100 (4): 497–506. https://doi.org/10.1007/s10482-011-9605-y.

Vergara-Álvarez, Israel, Francisco Quiroz-Figueroa, María Concepción Tamayo-Ordóñez, Amanda Alejandra Oliva-Hernández, Claudia Patricia Larralde-Corona, and José Alberto Narváez-Zapata. 2019. "Flocculation and Expression of *FLO* Genes of a *Saccharomyces cerevisiae* Mezcal Strain with High Stress Tolerance." *Food Technology and Biotechnology* 57 (4): 544–53. https://doi.org/10.17113/ftb.57.04.19.6063.

Waleckx, Etienne, Anne Gschaedler, Benoît Colonna-Ceccaldi, and Pierre Monsan. 2008. "Hydrolysis of Fructans from *Agave tequilana* Weber Var. Azul during the Cooking Step in

a Traditional Tequila Elaboration Process." *Food Chemistry* 108 (1): 40–48. https://doi.org/10.1016/j.foodchem.2007.10.028.

Walker, Graeme M., Patricia Lappe-Oliveras, Rubén Moreno-Terrazas C., Manuel Kirchmayr, Melchor Arellano-Plaza, and Anne Christine Gschaedler-Mathis. 2019. "Yeasts Associated with the Production of Distilled Alcoholic Beverages." In *Yeasts in the Production of Wine*, 477–512. New York, NY: Springer New York. https://doi.org/10.1007/978-1-4939-9782-4_16.

Zizumbo-Villarreal, Daniel. 1996. "History of Coconut (*Cocos nucifera* L.) in Mexico: 1539-1810." *Genetic Resources and Crop Evolution* 43 (6): 505–15. https://doi.org/10.1007/bf00138827.

Zizumbo-Villarreal, Daniel, Fernando González-Zozaya, Angeles Olay-Barrientos, Rafael Platas-Ruíz, Mariza Cuevas-Sagardí, Laura Almendros-López, and Patricia Colunga-GarcíaMarín. 2009. "Archaeological Evidence of the Cultural Importance of *Agave spp*. In Pre-Hispanic Colima, Mexico." *Economic Botany* 63 (3): 288–302. https://doi.org/10.1007/s12231-009-9092-5.
